# Probabilistic Carbon Analysis of Pakistan’s Bridges Unveils the Urgent Needs of Overdesign Optimization and Policy Transformation

**DOI:** 10.34133/research.1175

**Published:** 2026-03-03

**Authors:** Hazib Hafiz Muhammad, Shabbir Imran, Khalid Hafiz Humza, Limao Zhang, Jianjun Qin, Yue Pan

**Affiliations:** ^1^ State Key Laboratory of Ocean Engineering, Shanghai Key Laboratory for Digital Maintenance of Buildings and Infrastructure, School of Ocean and Civil Engineering, Shanghai Jiao Tong University, Shanghai 200240, China.; ^2^ Prime Engineering & Testing Consultants (Pvt) Ltd., Islamabad, Pakistan.; ^3^ School of Civil and Hydraulic Engineering, National Center of Technology Innovation for Digital Construction, Huazhong University of Science and Technology, Wuhan 430074, Hubei, China.

## Abstract

Rapid infrastructure expansion in emerging economies is increasing construction-related carbon emissions. Using a probabilistic assessment of 52 planned bridges on Pakistan’s M-13 Motorway, the average carbon emission intensity (CEI) is 1,430 kg CO_2_eq per square meter, 1.15 to 1.50 times higher per m^2^ of deck area than international benchmarks. The raw material (extraction and production) phase dominates the footprint (94.4%), with reinforcement (48.9%) and concrete (39.4%) as the principal material contributors; both display high variability (coefficients of variation 67% to 130%), signaling substantial uncertainty. Evidence points to systemic inefficiencies, including conservative overdesign, reliance on foreign codes, and the absence of standardized local emission data. Targeted measures, structural optimization using digital tools, wider adoption of recycling and low-carbon mixes, and development of context-specific emission factor databases offer a scalable pathway to lower-carbon bridge infrastructure in Pakistan and comparable settings.

## Introduction

The architecture, engineering, and construction (AEC) industry is a substantial contributor to global environmental challenges, accounting for over 40% of global energy consumption and nearly 40% of anthropogenic carbon emissions [1,2]. Concrete infrastructure alone inflicts over USD 300 billion in annual climate and health damages, posing substantial barriers to global decarbonization efforts [3]. Within this sector, infrastructure systems, particularly transportation networks, constitute a major share of emissions due to the carbon-intensive nature of material production, construction activities, and long-term operational demands [4–7]. With global infrastructure investments projected to exceed $90 trillion by 2030, the integration of sustainable design principles has become an urgent necessity rather than a discretionary practice [8]. Notably, efficiency gains in the built environment alone could cut U.S. energy-related CO_2_ by 6% to 11% and PM_2.5_ by 18% to 25% by 2050 [9,10]. Simultaneously, as global temperatures rise, many regions, including Pakistan, are encountering conditions that exceed the “human climate niche”, a range of environmental conditions historically conducive to human health and well-being [11]. This shift poses significant risks to human health, labor productivity, and overall societal stability, underscoring the imperative for climate-resilient infrastructure planning and development [11,12].

In Pakistan, rapid urbanization and economic growth have driven unprecedented infrastructure investment, with bridge construction emerging as a cornerstone of transportation expansion [13]. While this development has enhanced connectivity and facilitated energy access, it has concurrently driven up per-capita emissions as communities adopt more energy-intensive services [14]. Yet, this progress comes at an environmental cost: The country’s reliance on foreign design codes, inconsistent material quality, and conservative engineering practices have led to systemic overdesign, a phenomenon where structures are built with excessive material reserves beyond safety requirements [15,16]. Consequently, these overdesigned structures inflate embodied carbon and constrain livelihood adaptation, highlighting their far-reaching social and environmental impacts [17]. Studies indicate that concrete mixtures in Pakistani bridges are overdesigned by 25% to 36%, mirroring trends observed in other developing economies [18]. As a result, material consumption surges, amplifying embodied carbon emissions and running counter to Pakistan’s Paris Agreement commitments [19]. Ultimately, achieving international climate targets hinges on decarbonizing infrastructure development at scale.

We hereby undertake the investigations on refined quantitative analysis of carbon emissions of Pakistan’s recent large-scale bridge construction to illustrate the problems in Pakistan’s engineering practice and further to identify the potential solutions. Bridge construction, widely distributed in recent years in Pakistan as a sign of rapid development, presents unique carbon accounting challenges due to structural complexity, site-specific design variations, and supply chain opacity. Unlike standardized buildings, bridges require customized foundations and reinforcement systems sensitive to terrain, river hydrology, and seismic conditions [20,21]. These factors intersect within Pakistan’s fragmented data landscape where localized emission factors are scarce, and environmental oversight operates in silos, mirroring the institutional fragmentation common to rapidly urbanizing Global South regions [22], and are further compounded by cross-border material sourcing and lifecycle inventories that lack sufficient granularity. Consequently, traditional carbon assessment methods face 3 critical limitations [23,24]. First, process-based life cycle assessments (LCAs) often exclude upstream emissions, such as those associated with imported steel, due to ambiguities in defining system boundaries [25,26]. Second, input–output (IO) models rely on broad sectoral averages that obscure the project-specific material intensities inherent to bridge construction [27,28]. Such aggregation has been shown to conceal critical regional trade-offs and erode local design flexibility in net-zero energy system planning [29]. Third, hybrid methods, though theoretically comprehensive, underperform in low-data contexts like Pakistan due to inconsistent reporting and reliance on unvalidated assumptions [30]. Together, these methodological constraints risk systematically underestimating carbon emissions from complex infrastructure projects, thereby undermining the effectiveness of decarbonization strategies and policy interventions. In light of these limitations, infrastructure case studies using Monte Carlo show that site conditions and operational choices can materially shift net emissions, reinforcing the need for location-specific, probabilistic accounting [31].

To overcome the limitations of conventional carbon accounting in data-sparse settings, this study proposes a probabilistic semiquantitative framework specifically designed for low-data environments. Traditional deterministic process-based LCA can be constrained by incomplete and nonuniform life cycle inventory (LCI) data and inconsistent system boundaries, while IO and hybrid approaches may rely on highly aggregated sector averages or require extensive harmonization that is difficult to achieve where local inventories are scarce [32]. These issues are particularly relevant in Pakistan; for example, cement-sector data remain limited and heterogeneous [33], and emission factors can vary substantially across plants due to differences in clinker content, kiln efficiency, and fuel mix, within a coal-intensive production context. Departing from deterministic LCAs, the proposed framework explicitly incorporates uncertainty along 2 critical dimensions. First, material emission factors are characterized using Monte Carlo simulations (MCSs) informed by global datasets, calibrated to reflect regional energy profiles, such as Pakistan’s coal-intensive cement production mix. Second, design variability is captured through sensitivity analyses of overdesign parameters, including excess concrete strength margins. To facilitate meaningful comparisons across projects and infrastructure types, we introduce carbon emission intensity (CEI) (e.g., kg CO_2_eq/m). Moreover, reliance on imported materials introduces additional variability in material pathways, further compounding life cycle emissions uncertainty. Overall, the framework provides a rigorous and adaptable methodology for enhancing the accuracy of carbon assessments in complex infrastructure systems.

This study advances infrastructure carbon accounting by integrating probabilistic modeling with region-specific data quality indicators (DQIs), offering a more nuanced and context-aware approach to emission estimation. However, existing infrastructure carbon assessments, particularly in data-scarce regions, often rely on deterministic point emission factors and do not systematically propagate parameter uncertainty or account for variability in data quality and imported material pathways, limiting the trustworthiness of their conclusions. By systematically addressing uncertainties in emission factors and material sourcing, the proposed framework enables comparative assessments of carbon efficiency across diverse infrastructure types and design configurations. Our investigations unveil the pressing need to recalibrate Pakistan’s engineering standards specifically to reduce dependency on prescriptive foreign codes, enhance the resolution and reliability of localized material emission databases, and implement transparent reporting mechanisms for imported construction materials. These policy transformations are critical to aligning national infrastructure development with global decarbonization goals while ensuring structural resilience in the face of regional environmental and geotechnical conditions.

## Results

### Comprehensive uncertainty analysis of carbon emissions factors

The M-13 Motorway Corridor (117 km, Kharian–Rawalpindi), as one of the latest critical transportation projects in Pakistan, is taken here for assessing life-cycle carbon emissions. Comprising 52 prestressed concrete (PSC) structures, 26 major bridges, and 26 overpass bridges, spanning varied geotechnical conditions, the project integrates advanced engineering solutions, including deep-pile river crossings and shallow floodplain foundations, and conforms to both national and international design standards. As Pakistan’s first large-scale motorway developed under a public–private partnership (PPP) and a build–operate–transfer (BOT) model, the M-13 exemplifies the adoption of modern construction technologies, innovative materials, and sustainability-focused design practices. The 52 PSC structures along the motorway employ a uniform superstructure of prestressed I-girders and a reinforced concrete (RC) substructure, with concrete and reinforcing steel being the principal material inputs. The carbon intensities associated with these materials are modeled not as deterministic values but as probabilistic distributions, aiming to capture variability in global supply chains and production processes.

**Fig. 1.**
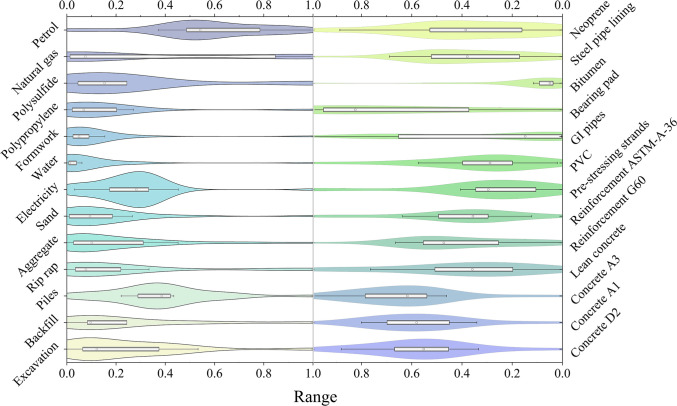
Distribution of CEFs across typical materials used in bridge construction. The plot illustrates the range, density, and spread of emission data for each material, highlighting uncertainty and variability across inputs. The range in the plot represents the CEFs of each material, normalized to a common scale for comparison (Data S1).

Figure 1 and Table 1 illustrate the empirical distributions of carbon emission factors (CEFs) across key bridge construction materials. The vertical span of each violin reflects the observed spread in emission factors for each material, enabling direct comparison of relative variability across inputs. Structural concrete exhibits relatively narrow normal distributions, with variability confined to ±20% around the mean, whereas reinforcing and prestressing steels follow broader log-normal distributions, indicative of greater uncertainty. For instance, Grade-60 reinforcing steel displays a 5th to 95th percentile range that is nearly 3 times the mean value (151.56 to 474.07 kg CO_2_eq/m^3^ for concrete D2; Table 1), whereas structural concrete demonstrates a relatively stable range of ±20% around the mean. This variability primarily reflects heterogeneity in production methods and energy sources, material specifications and quality control, transport logistics and haul distances, on-site equipment deployment, and the system boundaries defined in the underlying inventories. These disparities underscore the impact of fragmented supply chains, inconsistent recycling practices, and regional manufacturing variations on material-level carbon footprints.

**Table 1.** Statistical result of carbon emission factors for construction materials (Supplementary References). The table presents the mean, SD, emission factor range, and 90% confidence interval (CI) for each material. Units vary by material type (e.g., per cubic meter, per kilogram, per meter, per liter, or per megajoule), reflecting the diverse sources and usage forms. These data support accurate carbon assessments and highlight the variability and uncertainty associated with each material’s emissions.

Material	Unit	Mean	SD	Carbon emission factor range	90% confidence interval (CI)
Concrete D2	kg CO_2eq_/m^3^	332.978	70.147	151.56–474.07	188.59–468.43
Concrete A1	kg CO_2eq_ /m^3^	280.542	55.575	132.8–392.91	155.13–398.37
Concrete A3	kg CO_2eq_ /m^3^	237.995	38.918	125.98–305.72	163.91–307.71
Lean concrete	kg CO_2eq_ /m^3^	209.552	74.561	103.2–365.51	114.89–323.22
Reinforcement G60	kg CO_2eq_ /kg	1.785	0.844	0.35–3.84	0.37–3.26
Reinforcement ASTM-A-36	kg CO_2eq_ /kg	2.517	0.676	1.38–4.16	1.41–3.83
Prestressing strands	kg CO_2eq_ /kg	2.189	1.309	0.66–5.64	0.7–4.65
PVC	kg CO_2eq_ /m	2.000	1.216	0.32–5.37	0.38–3.62
GI pipes	kg CO_2eq_ /m	0.273	0.279	0.0088–0.84	0.02–0.67
Bearing pad	kg CO_2eq_ /cm^3^	0.006	0.003	0.00197–0.007847	0–0.01
Bitumen	kg CO_2eq_ /kg	0.085	0.086	0.0375–0.45	0.02–0.21
Steel pipe lining	kg CO_2eq_ /m	1.694	0.813	0.35–3.91	0.35–3.05
Neoprene	kg CO_2eq_ /kg	0.016	0.010	0.0013–0.0393	0.01–0.04
Diesel	kg CO_2eq_ /L	2.621	0.628	0.41–3.24	1.4–3.86
Excavation	kg CO_2eq_ /m^3^	3.976	4.141	0.024–16.65	0.32–10.57
Backfill	kg CO_2eq_ /m^3^	3.146	3.868	0.0175–13.62	0.3–8.9
Piles	kg CO_2eq_ /m	108.071	38.834	38–222.5	40.92–175.86
Rip rap	kg CO_2eq_ /m^3^	8.663	8.277	3.11–28.68	3.31–24.87
Aggregate	kg CO_2eq_ /m^3^	9.928	10.571	1.27–45.9	2.47–29.31
Sand	kg CO_2eq_ /m^3^	8.782	10.964	0.8–51.8	1.25–27.11
Electricity	kg CO_2eq_ /MJ	0.194	0.122	0.0016–0.74	0.03–0.41
Water	kg CO_2eq_ /kg	0.003	0.006	0.0001–0.031	0–0.02
Formwork	kg CO_2eq_ /m^2^	5.092	8.862	0.33–45.7	0.38–22.06
Polypropylene	kg CO_2eq_ /kg	3.078	2.759	1.34–12.72	0.68–7.99
Polysulfides	kg CO_2eq_ /kg	1.753	1.801	0.41–5.7	0.38–5.27
Natural gas	kg CO_2eq_ /MJ	0.291	0.298	0.0513–0.75	0.02–0.67
Petrol	kg CO_2eq_ /L	2.438	0.418	1.43–3.16	1.58–3.14

These results yield 2 critical insights. First, the carbon footprint of infrastructure systems is intrinsically uncertain, underscoring the need for assessment frameworks that explicitly quantify uncertainty to avoid systematic underestimation of emissions. Second, the greatest sources of variability in carbon estimates stem from materials embedded within globalized supply chains, such as steel and polymers, whereas locally sourced aggregates such as sand and gravel exhibit relatively stable and predictable emission profiles. This pronounced uncertainty, particularly in emission factors for globally sourced materials, highlights a critical data gap, characterized by a lack of localized emission databases and systematic emissions monitoring in Pakistan, which significantly amplifies uncertainties in carbon accounting. Enhancing the precision and reliability of carbon accounting in large-scale infrastructure projects therefore requires targeted interventions, including improved supply chain transparency, the strategic substitution of high-uncertainty materials, and the development of regionally calibrated emission-factor databases.

### Refined quantitative analysis of carbon emissions

Definition of structural characteristics, including height, pier height, and foundation complexity, plays a crucial role in the carbon emissions of bridge and overpass construction. Taller and more intricate structures, for example, necessitate greater material volumes, thereby increasing embodied carbon. The majority of emissions arise during the raw material extraction and production phase, a stage characterized by considerable variability. MCSs yield a total emission mean of 204.01 $\times 10^{6}$ kg CO_2_eq, with a 90% confidence interval ranging from 114.04 $\times 10^{6}$ to 293.80 $\times 10^{6}$ kg CO_2_eq, highlighting substantial variability in the results. A detailed sample calculation for a representative bridge is provided in Data S2.

**Fig. 2.**
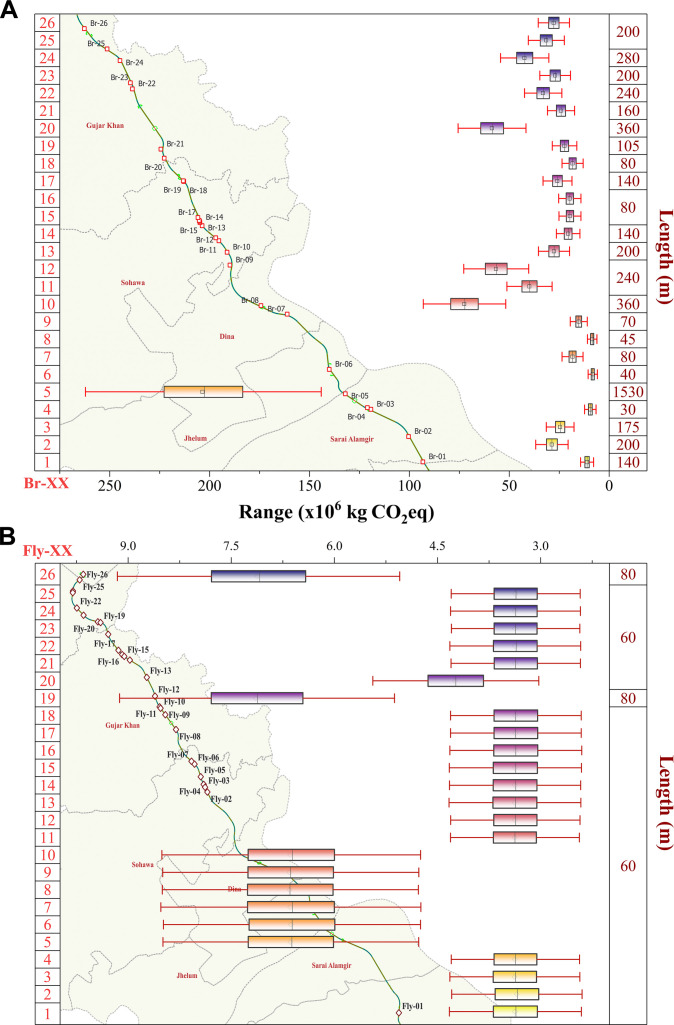
Carbon emission distribution of bridges along the project route. (A) Map and boxplot showing the life cycle carbon emissions (kg CO_2_eq) of bridges along the M-13 Motorway from Kharian and Rawalpindi. Each bridge (Br-01 to Br-26) is marked on the route map. (B) Map and boxplot showing the life cycle carbon emissions (kg CO_2_eq) of overpass bridges along the M-13 Motorway from Kharian to Rawalpindi. Each overpass bridge (Fy-01 to Fy-26) is marked on the route map. Boxplots display emission ranges with corresponding bridge lengths (in meters), and red error bars indicate uncertainty (Table S1).

Carbon emissions of bridges are heavily influenced by structural characteristics. For instance, Bridge-11 and Bridge-12 are both 240 m in length; however, their carbon emissions differ significantly, with Bridge-11 having a mean emission of 40 $\times 10^{6}$ kg CO_2_eq and Bridge-12 rising to 57 $\times 10^{6}$ kg CO_2_eq. This increase can be attributed to Bridge-12’s greater height, which requires additional construction materials. Similarly, Bridge-19 and Bridge-20, though identical in length (240 m), show a marked difference in emissions, 22.4 $\times 10^{6}$ kg CO_2_eq for Bridge-19 versus 58.7 $\times 10^{6}$ kg CO_2_eq, suggesting that foundation depth and pier design significantly impact embodied carbon. These emission variations are visually illustrated in Fig. 2A, which highlight how structural features beyond length contribute to carbon intensity.

For overpass bridges, i.e., flyovers (Fly), carbon emissions follow a similar trend, where features such as length, pier height, and the number of lanes significantly impact the total emissions. For example, Fly-19 and Fly-20, both 60 m in length, demonstrate different emission profiles due to variations in their design (see Fig. 2B). Fly-19, which includes more lanes and a higher pier height, has a mean emission of 71.5 $\times 10^{6}$ kg CO_2_eq, whereas Fly-20, with fewer lanes and a lower pier height, shows a lower mean emission of 42.5 $\times 10^{6}$ kg CO_2_eq. This trend is further illustrated by Fly-12 and Fly-15, both 60 m long, yet differing in carbon intensity: Fly-12 generates 33.8 × 10^6^ kg CO_2_eq, while Fly-15 reaches 42.4 × 10^6^ kg CO_2_eq. All flyovers on the M-13 corridor are 2-lane structures with a uniform pier height of 7 m, except Fly-19 (22-m deck length) and Fly-26 (15.5-m deck length). These structural parameters confirm that substructure height and superstructure span, even with consistent lane capacity, significantly influence embodied carbon variability. These disparities underscore that structural parameter beyond horizontal length, particularly pier height and deck width, play a dominant role in shaping the carbon footprint of overpass bridges. The findings reinforce the need for emissions accounting frameworks that incorporate design-specific attributes rather than relying solely on length-based estimation methods.

While such differences may be structurally justified, the degree of variation, particularly among bridges with similar functional requirements, suggests that material usage is not always proportionally aligned with design necessity. This raises the possibility that conservative safety factors due to a lack of a knowledge base or insufficient site investigations, limited use of optimization tools, or lack of performance-based design approaches may contribute to excessive material specifications in some cases. As such, these patterns may indicate instances of structural overdesign [34], a consideration further examined in the discussion.

**Fig. 3.**
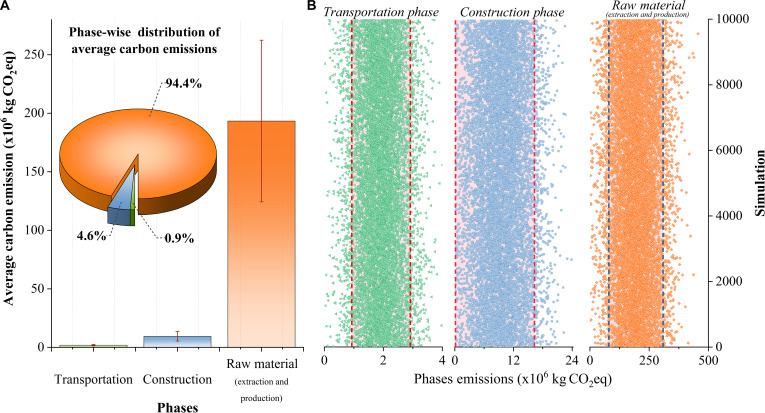
Phase-wise distribution of average carbon emissions for the M-13 Motorway project. (A) Bar and pie charts show that raw material (extraction and production) is the dominant emissions source (94.4% of total, 193.25 × 10^6^ kg CO_2_eq), with construction and transportation contributing much less. Error bars reflect variability from MCSs. (B) Monte Carlo scatterplots display the range of simulated emissions for each phase over 10,000 runs. Vertical dashed lines indicate the 5th and 95th percentile values, highlighting the spread and uncertainty in each phase (Data S3).

Carbon emissions of bridge construction can be evaluated across 3 key activity phases: raw material (extraction and production), transportation, and construction (Fig. 3A). Among these, the raw material (extraction and production) phase emerges as the dominant contributor, accounting for approximately 94.4% of total emissions. MCSs estimate a mean emission of 193.25 $\times 10^{6}$ kg CO_2_eq for this phase, with a wide uncertainty range spanning from 69.38 $\times 10^{6}$ to 293.80 $\times 10^{6}$ kg CO_2_eq and an SD of 68.69 $\times 10^{6}$ kg CO_2_eq, underscoring its critical role in overall emission variability. The construction phase generates 9.45 $\times 10^{6}$ kg CO_2_eq on average, which accounts for 4.6% of total emissions. The assessed CO_2_eq emissions for this phase range from 4.22 $\times 10^{6}$ kg CO_2_eq to 16.94 $\times 10^{6}$ kg CO_2_eq, with an SD of 4.17 $\times 10^{6}$ kg CO_2_eq, indicating better emission control than in the raw material (extraction and production) phase. The transportation phase accounts for only 0.9% of the total emissions. Emissions from transportation are notably lower, with an average emission of 1.91 $\times 10^{6}$ kg CO_2_eq, ranging from 0.59 $\times 10^{6}$ kg CO_2_eq to 2.64 $\times 10^{6}$ kg CO_2_eq, and a slight SD of approximately 0.60 $\times 10^{6}$ kg CO_2_eq, reflecting relatively low uncertainty in the transportation emissions. Another perspective on emission uncertainty is afforded by Fig. 3B, which reveals the extent of variability within each activity phase. The width of the distribution for each phase illustrates the relative spread of simulated outcomes, with broader distributions indicating greater uncertainty. Notably, the raw material (extraction and production) phase exhibits the widest spread, while the construction and transportation phases display comparatively narrower distributions.

**Fig. 4.**
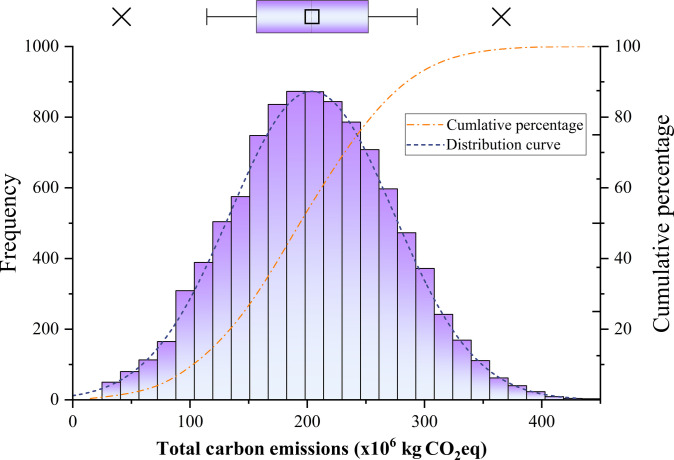
MCS results for the total carbon emissions of the project (in kg CO_2_eq). The histogram shows the frequency distribution of emissions, centered around a mean of 204.01 $\times 10^{6}$ kg CO_2_eq, with a 90% confidence interval ranging from 114.04 $\times 10^{6}$ kg CO_2_eq to 293.80 $\times 10^{6}$ kg CO_2_eq. The overlaid boxplot highlights the variability and spread of the data, while the cumulative percentage curve and distribution curve illustrate the probabilistic behavior of emission outcomes (Data S3).

Using MCSs, the analysis yields a mean estimate of 204.0 × 10^6^ kg CO_2_eq for total embodied carbon emissions, with values ranging from a minimum of 1.56 $\times 10^{6}$ kg CO_2_eq to a maximum of 447.24 $\times 10^{6}$ kg CO_2_eq. The 90% confidence interval spans from 114.04 $\times 10^{6}\;$ to 293.80 $\times 10^{6}$ kg CO_2_eq, highlighting substantial variability in the emission estimates. This uncertainty in emissions is further illustrated (Fig. 4)., which presents the full distribution of stochastic simulation outputs, offering a comprehensive visual depiction of emission variability across the project scope.

### Environmental impact assessment

**Fig. 5.**
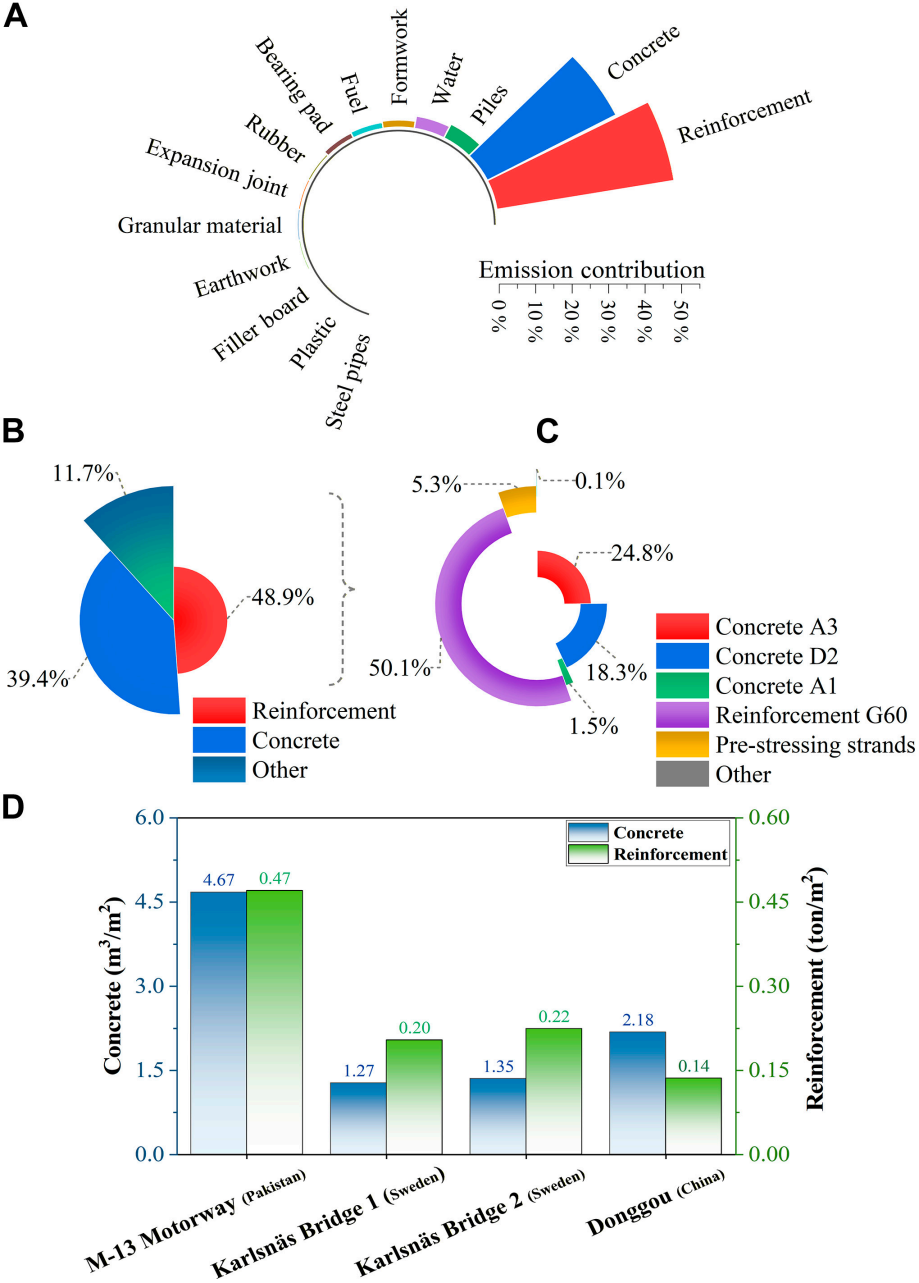
Material-wise and type-wise carbon emissions in the M-13 motorway project and comparative material intensity per unit area. (A) Project-wide material inventory by embodied carbon share shows concrete and reinforcement as the dominant contributors. (B) Dominant contributions of reinforcement and concrete, with all other materials aggregated. (C) Detailed breakdown of individual concrete and reinforcement types. (D) Comparison of concrete and reinforcement intensity per unit area for the M-13 Motorway and 3 reference concrete bridges (Karsalan Bridge 1, Karsalan Bridge 2, and Donggou) (see Table S22).

The environmental impact assessment of the M-13 Motorway project, as illustrated in Fig. 5A to C, reveals that concrete and reinforcement materials are the principal sources of carbon emissions, collectively accounting for 88.3% of the project’s total emissions. Specifically, 48.9% of the emissions are attributed to reinforcement, while 39.4% are from concrete and the remaining 11.7% is contributed by other materials (Fig. 5B). This underscores the dominant role of concrete and reinforcement in the carbon footprint of large-scale infrastructure. A further breakdown (Fig. 5C) shows that within the concrete and reinforcement category, G60 reinforcement is the largest single contributor, responsible for 50.1% of emissions. Concrete A3 and concrete D2 follow, contributing 24.8% and 18.3%, respectively. Prestressing strands account for 5.3%, while concrete A1 adds another 1.5%. Other concrete and reinforcement types contribute a negligible 0.1% overall. The comparative assessment of material intensity (Fig. 5D) reveals that the M-13 Motorway utilizes substantially greater quantities of both concrete and reinforcement per unit length of structure than the 3 international reference bridges. Specifically, the motorway section consumes approximately 4.67 tons of concrete and 0.47 tons of reinforcement per square meter, compared to 1.27 to 1.35 tons of concrete and 0.20 to 0.22 tons of reinforcement for Karsalan Bridges 1 and 2, and just 2.18 tons of concrete and 0.14 tons of reinforcement per square meter for the Donggou Bridge. These stark differences underscore not only the disproportionate contribution of specific material categories to total carbon emissions but also a pronounced dependence on primary, carbon-intensive resources, with minimal integration of recycled or low-carbon alternatives. Consistent with Figs. 3 and 5, model sensitivity concentrates on raw material drivers, i.e., reinforcement and concrete emission factors and quantities, which together explain the bulk of output variability; transportation and construction terms contribute comparatively little to variance at the project scale.

This elevated material demand may reflect heightened structural or durability requirements in the M-13 context; however, the magnitude of excess suggests a broader tendency toward material overuse. This is likely exacerbated by conservative design practices, limited use of optimization tools, and an absence of effective frameworks for sustainable material substitution. The continued reliance on conventional high-carbon cement formulations and the low adoption of innovative, low-impact materials further amplify the project's embodied carbon footprint. In parallel, typology also matters: Different bridge types carry different material intensities and fabrication routes, steel/composite systems are typically higher upfront, PSC is intermediate, and RC slabs tend to be lower, although high piers and heavy foundations can outweigh these typology differences. Collectively, these patterns highlight systemic inefficiencies in material utilization and emphasize the urgent need to improve material efficiency in future infrastructure developments.

### CEI of transportation infrastructures

This study addresses the challenge of benchmarking carbon efficiency by analyzing CEI, measured as emissions per functional output of deck area (i.e., kg CO_2_eq/m^2^). The analysis covers 4 international transportation infrastructure case studies alongside the M-13 Motorway. To make a fair comparison, this study focuses on RC structures. The benchmark set includes 4 international transportation infrastructure case studies selected to enable like-for-like comparison with RC structures; these include the Karlsnäs Bridges in Sweden [35], the Donggou Bridge in China [36], the Tuochuan Tunnel in Jiangxi, China [37], and the M-13 Motorway in Pakistan. This comparative approach enables a more nuanced understanding of carbon efficiency across diverse transportation infrastructure systems. To address the benchmarking challenge, this study explicitly distinguishes between structurally comparable and structurally dissimilar reference projects. The primary benchmarks for the M-13 bridges are the reinforced and PSC highway bridges (Karlsnäs Bridges 1 and 2, and the Donggou Bridge), which are closest in terms of functional role, span range, deck width, and dominant material composition. Other international cases, such as the Tuochuan Road Tunnel and the Quincy Bayview steel cable-stayed bridge, are included only as contextual reference points to illustrate the wider CEI-cost space; they are not treated as like-for-like structural comparators because their excavation volume, structural systems, and material intensity profiles differ fundamentally from surface RC/PC (prestressed concrete) highway bridges.

**Fig. 6.**
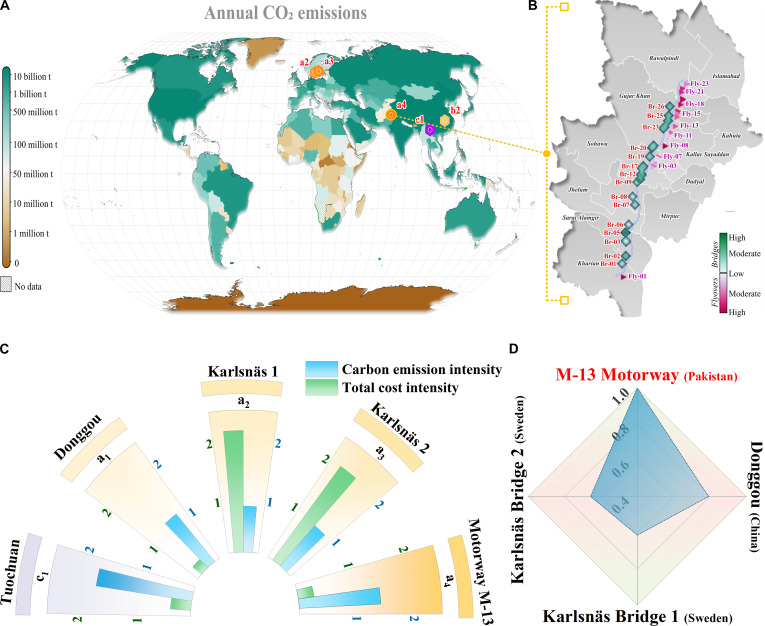
Comparison of CEI and total cost intensity between the RC bridges of the M-13 Motorway project (Pakistan) and the counterparts in other countries. (A) Global map displaying annual CO_2_ emissions for 2023 by country, highlighting the geographical distribution of carbon outputs with particular emphasis on regions containing major infrastructure projects [83]. (B) Regional map of the M-13 Motorway project, illustrating the spatial distribution of carbon intensive bridges (Br) and overpass bridge (Fly) within the study area. (C) Multi-dimensional benchmarking of CEI (×10^3^ kg CO_2_eq/m^2^) and total cost intensity (×10^3^ $/m^2^) of the M-13 Motorway project against global transport infrastructure (Table S3). (D) Comparison of CEI of those RC bridges [the results of M-13 Motorway (Pakistan) are scaled to 1].

A comparative analysis of the M-13 Motorway’s environmental performance, as presented in Fig. 6, benchmarks its CEI and cost efficiency against diverse infrastructure typologies, including RC bridges and tunnels. The results reveal that the M-13 Motorway exhibits significantly higher CEI than its global counterparts, alongside substantial cost inefficiencies. For instance, while projects such as the Donggou Bridge and the Tuochuan Tunnel demonstrate optimized carbon performance relative to their scale and functional requirements, the M-13 project’s emissions far exceed these benchmarks. This discrepancy highlights underlying inefficiencies in material utilization, shortcomings in construction methodologies, and vulnerabilities in supply chain sustainability across Pakistan’s infrastructure sector. The project exhibits a CEI of $1.43\times 10^{6}$ kg CO_2_eq/m^2^, coupled with an associated cost of approximately USD 0.28 million per square meter. In comparison, RC bridges such as Karlsnäs Bridge 1 and Karlsnäs Bridge 2 show lower emission levels of $0.88\times 10^{6}$ kg CO_2_eq/m^2^ and $0.94\times 10^{6}$ kg CO_2_eq/m^2^, respectively. The Donggou Bridge demonstrates the highest carbon efficiency among the analyzed structures, with a CEI of only $1.13\times 10^{3}$ kg CO_2_eq/m^2^, while the Tuochuan Tunnel records a moderately higher value of $1.72\times 10^{3}$ kg CO_2_eq/m^2^. By contrast, the Quincy Bayview Bridge (USA; steel superstructure) reports a CEI of $0.67\times 10^{3}$ kg CO_2_eq/m^2^ but a notably higher cost intensity of USD 3.94 million per square meter, indicating that lower carbon intensity does not necessarily correspond to lower expenditure. From the detailed material breakdown and sample bridge calculations, it is evident that the superstructure, particularly the PSC girders and 6-lane deck configuration, constitutes the most carbon-intensive component, while substructure variation (e.g., pier height and foundation type) further explains part of the CEI difference across bridges. However, differences in geotechnical conditions (e.g., deep alluvial foundations on the M-13 corridor versus shallower or rock foundations in some benchmarks), pier height, and local seismic and hydraulic demands limit a strict one-to-one comparison of CEI values. The international cases are therefore interpreted as defining a plausible efficiency band rather than exact design targets for the M-13 bridges.

These findings are based on quantitative comparisons between the M-13 inventory and international bridge datasets (Karlsnäs, Donggou, Tuochuan, and Quincy Bayview). The comparison indicates that Pakistan’s elevated emission intensity stems primarily from uncoordinated policy specifications and fragmented data governance rather than structural necessity. Table 2 maps design, material, and procurement choices to their respective carbon consequences, illustrating how inconsistent specifications amplify material quantities and emission factors. The wide gap between the M-13 Motorway and its international counterparts underscores systemic inefficiencies arising from policy fragmentation and highlights the need to integrate carbon-cost optimization within Pakistan’s infrastructure planning framework.

**Table 2.** Policy-related practices and gaps. Summary of current approaches and associated CO_2_ implications.

Area	What the project used	What is missing/contradictory	How this raised CO_2_
Design code	Foreign code w/o local calibration	No local load factors	Higher rebar/cover → more steel
Concrete spec	No SCM/low-carbon requirement	No EPD/SCM clause	High-clinker mixes → more CO_2_
Procurement	Lowest price only	No carbon criterion	No incentive to pick low-EF suppliers
Recycling	No recycled content target	No rebar/asphalt RAP spec	All-virgin materials
EF data	Generic averages	No localized EF database	Conservative EFs

## Discussion

### Key driver of carbon inefficiency in Pakistan’s large-scale bridge

We critically evaluate the carbon efficiency of the M-13 Motorway bridges using a probabilistic LCA. Our investigations address the carbon inefficiencies in Pakistan’s large-scale bridge construction. The M-13 Motorway project’s CEI is evaluated as $1.43\times 10^{3}$kg CO_2_eq/m^2^ on average, which significantly exceeds those of the counterparts (RC bridges) in other countries. Our investigations show that the extraction and production of raw materials constitute the predominant sources of emissions, with concrete (concrete A3, concrete D2, and concrete A) and reinforcement steel (reinforcement G60 and prestressing strands) accounting for 42.67% and 48.67% of total emissions, respectively. Building on this, the probabilistic Monte Carlo analysis confirms that the same materials also dominate the uncertainty in total emissions. Perturbations of G60 reinforcement and structural concretes (A3 and D2) together explain the vast majority of output variance, mirroring their combined 88% share of mean embodied emissions. G60 reinforcement alone is responsible for roughly half of both mean emissions and modeled variance, while uncertainties in concrete emission factors and quantities account for most of the remaining spread. This concentration of impact and uncertainty in a small group of materials indicates that targeted interventions on structural steel and concrete will deliver far greater carbon reductions than diffuse, nonspecific mitigation efforts. These results collectively underscore the material-intensive character of current infrastructure design paradigms in Pakistan. These material intensity insights point toward embedded inefficiencies across the design, procurement, and construction spectrum, particularly in the form of excess reliance on high-carbon materials.

This disparity is reinforced by international evidence showing that in developing countries, engineers normally work with overly conservative design assumptions, frequently borrowed from foreign codes without local calibration. For instance, UN-Habitat (2018) [38] observed that low-income countries adopting foreign building standards often “set the bar too high”, creating unnecessary material demands and stifling local innovation. Similarly, Allwood et al. [39] reported that structural engineers often overspecify steel by 30% to 40%, with actual utilization in beams averaging only 50%, which is a result of unjustified safety margins. In Pakistan, similar trends are visible; the Khairabad Bridge, for example, was constructed with excess concrete strength due to outdated design practices and limited regulatory oversight [18]. Such misalignments between structural demand and material usage reflect the persistent issue of overdesign, contributing significantly to embodied carbon inefficiency. This pattern is quantitatively supported by the current study’s utilization ratios across 52 bridges on the M-13 corridor, where average utilization ratios of 0.85 to 0.90 for decks and 0.80 to 0.85 for girders contrast with lower values of 0.70 to 0.80 for piers and foundations, confirming a systemic tendency toward conservative substructure design and material overuse. Structural utilization ratios for representative bridges and flyovers on the M-13 corridor are given in Table S4.

Additionally, despite introducing performance-based principles in the 2021 Building Code of Pakistan (BCP-2021), the continued use of conventional seismic methods, such as the equivalent lateral force (ELF) procedure, exacerbates material inefficiencies by inflating design loads and failing to account for nonlinear performance [40]. These practices suggest a lag in regulatory modernization and coordination, leading to design choices that may be structurally conservative but environmentally and economically inefficient. This observation links directly to policy fragmentation, as the second key finding, wherein engineering standards are still rooted in foreign codes (e.g., AASHTO-LRFD and ASHRAE) not fully adapted to Pakistan’s geotechnical, climatic, or economic realities [41,42]. Recent analyses show that Pakistan’s Energy Conservation Building Code 2023 continues to import standards and material specifications without sufficient localization, and enforcement remains weak, a systemic issue highlighted by both academia and industry.

Compounding these structural and policy issues is the lack of localized environmental data. The present study demonstrates that the largest uncertainty in carbon estimates stems from carbon-intensive materials such as concrete and steel, products embedded in complex, globalized supply chains. These uncertainties were quantified using MCSs, which revealed wide variability. Pakistan currently lacks an official emissions factor database for construction materials; as a result, assessments rely heavily on international averages or outdated estimates. Beyond global totals, geographically resolved studies show that damages from short-lived co-pollutants depend strongly on where emissions occur, strengthening the case for location-specific carbon accounting and policy design [43]. For example, Pakistan’s cement production emissions have not been publicly tracked beyond 2012 [44], and the national greenhouse gas (GHG) inventory relies heavily on Intergovernmental Panel on Climate Change (IPCC) default factors. This data scarcity impedes reliable carbon accounting and limits the effectiveness of low-carbon strategies. These limitations form the basis of the third finding: data gaps, particularly the absence of regionally validated LCI data and the lack of a national emissions monitoring framework.

**Fig. 7.**
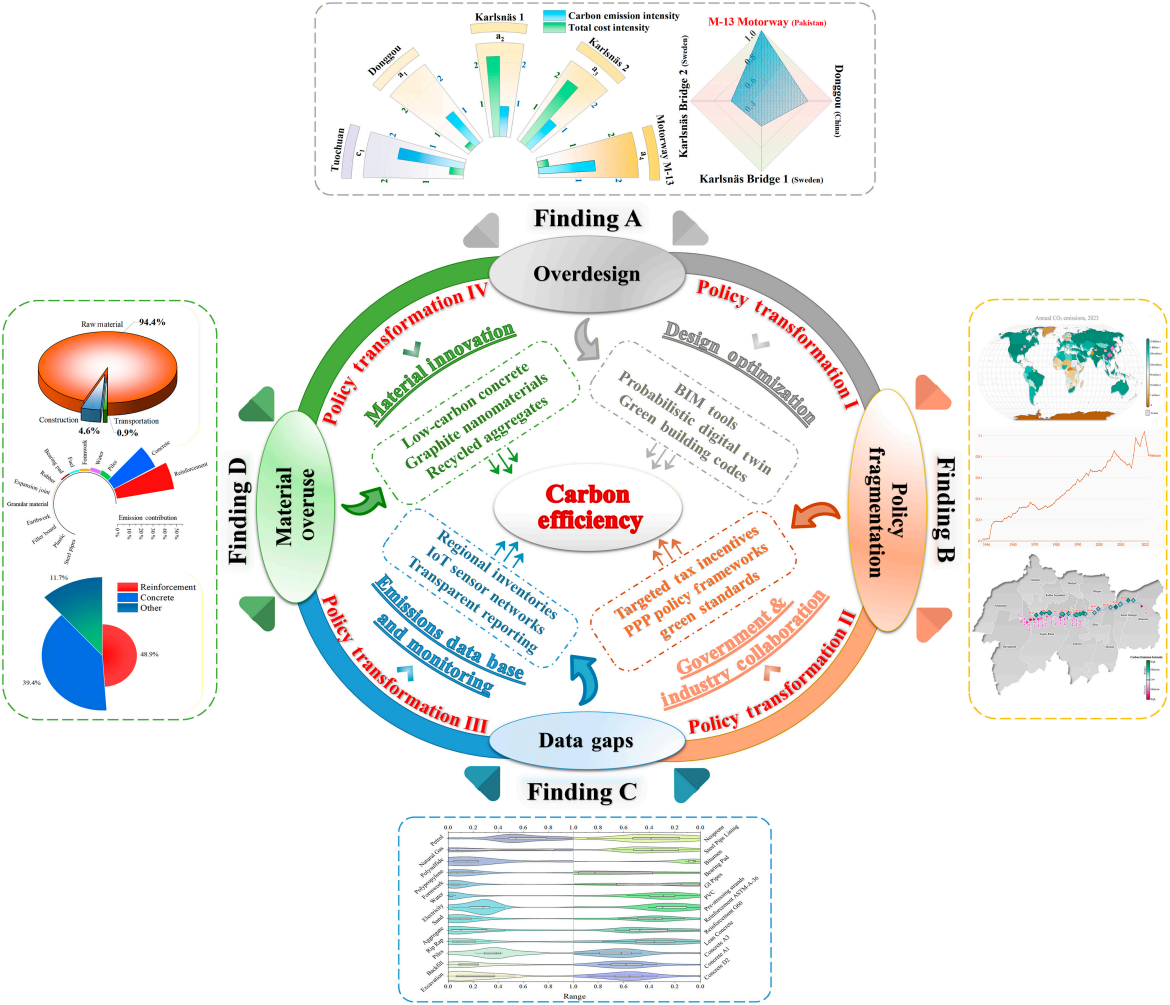
Policy-oriented strategies for sustainable infrastructure development, emphasizing material innovation incentives, optimized design policies, comprehensive emissions monitoring frameworks, and strengthened collaborative governance through industry–government partnerships.

Finally, the environmental assessment of the M-13 Motorway revealed a consistent pattern of elevated material intensity in the M-13 Motorway relative to international benchmarks, with noticeably higher volumes of concrete and reinforcement deployed per meter of structure. This trend suggests that improving material efficiency could be a priority for subsequent design iterations and procurement guidance. While some degree of variation may be attributed to structural or geotechnical factors, the magnitude and consistency of excess suggest that material use in M-13 is not proportionally aligned with functional necessity. Compounding this issue, the use of supplementary cementitious materials or recycled aggregates remains negligible in Pakistan’s infrastructure sector [42,45]. Studies and policy reviews confirm that green concrete options and alternative masonry units (e.g., fly-ash bricks) are underutilized, and that construction practices still largely depend on virgin materials sourced through inefficient methods [46]. Taken together, the comparative assessment strongly affirms the fourth and final key finding: material overuse, which characterizes the prevailing material practices observed in the M-13 Motorway project. Based on the semi-probabilistic life cycle assessment, we can conclude that these 4 interrelated findings (problems): (a) overdesign, (b) policy fragmentation, (c) data gaps, and (d) material overuse, collectively define the primary drivers of embodied carbon inefficiency in Pakistan’s large-scale bridges (see Fig. 7). The subsequent section will present 4 corresponding intervention streams, each directly addressing one of these findings, to establish a coherent, high-impact roadmap toward low-carbon infrastructure.

### Potential policy transformation in support of sustainability and carbon efficiency for Pakistan’s infrastructures

The probabilistic analysis revealed that uncertainties in concrete and steel emission factors accounted for the largest variability in total emissions (ranging from 67% to 130% of the mean). This quantitative evidence highlights where policy intervention is most urgently needed, specifically in improving data reliability, emission factor standardization, and design optimization. Building on these findings, the broader policy context in Pakistan reveals several systemic barriers that continue to impede low-carbon infrastructure development. Foremost among these are insufficient preconstruction environmental assessments and regulatory frameworks that fail to incorporate carbon accounting principles. Furthermore, the lack of standardized, policy-driven educational programs aiming to promote sustainable construction practices constrains capacity building across the sector. These policy gaps are compounded by fragmented supply chains and outdated regulatory instruments, which together inhibit the mainstream adoption and enforcement of low-carbon strategies. Additionally, uncertainties in the emission factors make the resulting total carbon emission estimates highly variable (from 67% to 130% of the mean), underscoring the policy challenges arising from the lack of trustworthy regional data management, unclear energy grid modes, and inconsistent material production methodologies. These gaps are summarized in a policy fragmentation matrix, which links each governance domain to its quantified impact on embodied carbon intensity along the M-13 corridor (Table 3). Addressing these issues, as emphasized by Meckling and Karplus [47] and Grassi et al. [48], requires the development of comprehensive, context-specific policies that standardize carbon quantification methods, ensure the reliability of regional data, and promote regulatory coherence across infrastructure sectors.

**Table 3.** Policy fragmentation matrix linking governance gaps to embodied carbon impacts along the M-13 corridor. The table summarizes key gap domains (design codes, material specifications, procurement practices, emission factor databases, and recycling), the Pakistan-specific issues and resulting inconsistencies with international best practice, the mechanisms through which these gaps increase embodied carbon, and their quantified impacts based on M-13 bridge data (e.g., utilization ratios, material intensities, CEI levels, and emission factor ranges).

Gap domain	Pakistan-specific issue	Key inconsistency/contradiction	Mechanism	Quantified carbon impact (M-13 data)​
Design codes	Foreign codes (e.g., AASHTO-LRFD) uncalibrated locally	BCP-2021 ELF seismic vs. imported ductility assumptions inflate loads	Overdesign in piers/foundations (utilization 0.70–0.80)	2–4× more concrete (4.67 t/m^2^ vs. 1.27–2.18 t/m^2^), 2–3× rebar (0.47 vs. 0.20 t/m^2^), CEI 1.43 × 10^3^ kg CO_2_eq/m^2^ (1.15–1.5× benchmarks)
Material specs	No SCM/recycled mandates	High-clinker mixes vs. international SCM/EPD standards	Locks in virgin high-EF materials	Concrete/rebar: 88.3% emissions (39.4% and 48.9%), 20–30% SCM could cut tens of 10^6^ kg CO_2_eq
Procurement	Price-only, no carbon criteria	Equivalent pricing ignores EF variations in suppliers	Favors high-EF cheap sources	EF variation 67–130% (steel/concrete), materials phase 94.4% total (193 × 10^6^ kg CO_2_eq)
EF databases	No local inventories, generic global	IPCC defaults vs. Pakistan coal-cement reality	Wide EF ranges, conservative assumptions	Concrete D2: 152–474 kg CO_2_eq/m^3^, G60 rebar: 0.35–3.84 kg/kg, total variability 67–130%
Recycling	No recycled content targets	Virgin-only vs. EU/Cross-rail mandates	Forfeits loop savings	Rebar ~50.1% emissions; recycled could cut ~18.6% CEI

Thus, the following policy transformation framework (see Fig. 7) is recommended to embed sustainability into infrastructure policies:

#### Potential policy transformation I: Material innovation

Quantitative analyses of the M-13 Motorway revealed that material overuse is the dominant source of embodied carbon inefficiency, with concrete and reinforcement quantities exceeding international benchmarks by roughly 20% to 30%. As highlighted in our environmental assessment, concrete and reinforcement collectively contribute to approximately 88.3% of total carbon emissions associated with infrastructure development; therefore, a 10% reduction in either their emission factors or their material demand would translate into an approximate 9% reduction in corridor-scale embodied carbon. This directly justifies the prioritization of low-carbon concrete and steel standards as core elements of the proposed policy transformation. Given this dominance, material innovation must be a central focus of policy interventions aimed at reducing the environmental footprint of the construction sector. Several studies show that policy instruments encouraging the use of recycled aggregates or low-carbon concrete could be associated with substantial emission reductions. Evidence from UK Crossrail indicates that a 50% cement replacement achieved an 18.6% reduction in carbon intensity [49]. In parallel, a graphene-enhanced concrete (“Concretene”) demonstration in Amesbury, UK, achieved 30% lower material usage and eliminated steel reinforcement [50].

To operationalize material innovation objectives, 4 policy levers could be considered for near-term implementation. One option is a minimum recycled content threshold for government-funded construction, potentially set at ≥20%, coupled with a sliding-scale tax rebate of up to 15% for projects that exceed 30% reuse. Secondly, public tendering procedures could incorporate bonus evaluation points for bids demonstrating a minimum of 25% supplementary cementitious materials or recycled aggregate, thereby guaranteeing demand and stimulating supplier capacity. Thirdly, national development banks could underwrite low-interest “green construction” loans, capped at 10% of project value, for adopters of validated low-carbon mixes such as Ground Granulated Blast Furnace Slag (GGBS) or fly-ash blends. Finally, landfill-fee reform could differentiate charges by waste stream, with higher fees for virgin-only concrete waste and rebates for source-separated recycled material, internalizing waste-management costs and rendering recycling economically advantageous. Collectively, these instruments may leverage existing institutional capacities and could facilitate broader adoption of recycled and low-carbon materials.

#### Potential policy transformation II: Design optimization

The persistent overdesign of concrete mixtures in Pakistani bridge projects represents a major source of embodied carbon inefficiency and highlights a significant opportunity for optimization. To address this inefficiency, national policies could incentivize, or where appropriate require, the adaptation of digital technologies such as building information modeling (BIM) and digital twin systems to optimize material use. International examples demonstrate the impact of such measures: BIM mandates have reduced steel usage by 25% in the Shanghai Tower [51] and At The Edge (Amsterdam), BIM-enabled smart systems deliver 70% lower electricity use than typical offices, and the building is net-energy-positive with a 98.36% BREEAM-NL score [52,53]. Similarly, probabilistic digital twin models have been shown to cut construction-related carbon emissions by up to 43% [54]. Capitalizing on these documented efficiencies, a staged BIM roadmap may be appropriate; for example, phased targets by 2025, 2027, and 2030 could support progressive capacity building.

Phase I (by December 2025): Mandate BIM level 1 on all public and private infrastructure projects exceeding PKR 5 million; designate 3 bridge and roadway pilot sites to validate model-based coordination benefits; and establish a foundational national BIM library containing standard structural and material components.
Phase II (by December 2027): Launch a “BIM Engineer Accelerator” in partnership with leading universities and the Higher Education Commission to certify 100 practitioners; integrate a compulsory BIM module into undergraduate civil engineering and architecture curricula; and introduce dual-tier national certifications for model coordination (level 1) and parametric design (level 2).
Phase III (by December 2030): Require BIM level 2 for all large-scale and high-risk infrastructure; codify digital twin asset management protocols within the Building Code of Pakistan; expand the BIM library to encompass MEP (mechanical, electrical, and plumbing) systems and sustainability metadata; and link permitting and payment milestones to verified BIM deliverables.

#### Potential policy transformation III: Government and industry collaboration

Pakistan’s fragmented regulatory environment and the absence of carbon-focused PPP guidelines have constrained coordinated action, limited industry participation, and deterred investment in low-carbon infrastructure solutions. To accelerate decarbonization, a unified policy architecture is needed to align governmental objectives with private-sector capabilities. Establishing clear national guidelines and incentive mechanisms for public–private partnerships can strengthen industry engagement, attract investment, and align private expertise with national carbon reduction goals. For example, the Green Deal “Het Nieuwe Draaien” has delivered 10% to 20% CO_2_ reductions through fuel switching and electrifying equipment, supported by national tax incentives (EIA, MIA/Vamil) [55,56]. Similarly, Singapore's BCA Green Mark Scheme, a regulatory-driven initiative, effectively increased recycled concrete use in projects such as the Punggol Digital District [57].

To address policy fragmentation, establishing a road sustainability partnership (RSP) could be explored as a coordination mechanism, bringing together the Ministry of Communications, the National Highway Authority (NHA), leading contractors, material suppliers, financiers, and research institutions. Under this arrangement, public–private partnership contracts could incorporate explicit carbon key performance indicators (KPIs), such as a cap on embodied carbon per lane-kilometer, and link milestone payments to verified emission outcomes. A dedicated 100-km Green Road Demonstration Corridor would serve as a living laboratory for recycled aggregate asphalt, BIM-driven alignment optimization, and on-site asphalt recycling, with all stakeholders continuously feeding real-time emissions data into a centralized emission factor registry. Complementary financial instruments, blended-finance guarantees, targeted tax rebates, and an annual “Green Road” award would reward contractors who exceed defined carbon efficiency benchmarks, while accredited certification bodies ensure rigorous, transparent verification. By integrating clear regulatory mandates, collaborative data platforms, and performance-based incentives, the RSP framework offers a coherent, scalable blueprint for sustainable highway networks across Pakistan.

#### Potential policy transformation IV: Emissions monitoring and reporting

Given the significant uncertainties in material emissions (67% to 130% of the mean) identified through probabilistic analysis, real-time emissions monitoring and transparent reporting system [e.g., using Internet of Things (IoT); blockchain-based registries] may improve data reliability. An illustrative case is the UK’s HS2 program. The government policy accelerated the use of IoT sensors and other environmental measures, contributing to an overall 33.8% reduction in carbon versus baseline [58,59]. Moreover, the European Emis-Chain project, backed by policy-level testing and integration, demonstrated significant capability in monitoring, reporting, and trading emissions from transportation sectors, thereby potentially enhancing transparency and accountability in carbon management [60]. Additionally, national strategies should prioritize developing region-specific carbon inventory databases. Systems such as Brazil’s SIDAC (Information System for Environmental Performance in Construction) have produced more representative LCA results than Ecoinvent by aligning data with domestic production contexts, while recent European studies revealed deviations of up to 17% in embodied carbon when regionalized background inventories were used instead of generic averages [61,62]. This demonstrates that localized databases are not merely supplemental tools but essential instruments for guiding targeted, context-aware carbon reduction policies. To address significant uncertainties in embodied carbon estimates and establish a transparent, data-driven emissions framework, a 3-phase roadmap could be considered over the next decade:

Phase I (2026 to 2028): Develop and validate a Pakistan-specific Emission Factor Database using domestic material and process data.
Phase II (2029 to 2030): Pilot IoT-enabled CO_2_ sensors on flagship projects and launch a cloud-based Emissions Registry with standardized calibration and reporting protocols.
Phase III (2031 to 2035): Integrate blockchain for immutable records, mandate real-time data submission with third-party audits, and introduce performance-based incentives (tax rebates, awards) to ensure compliance and drive continuous improvement.

The proposed policy transformations are designed in phased, evidence-based stages reflecting Pakistan’s current institutional and financial realities. Each recommendation builds upon proven international precedents, for example, the UK Crossrail’s material reuse targets, Singapore’s BCA Green Mark, and the HS2 and Emis-Chain digital monitoring systems, demonstrating that similar transitions have been technically and economically achievable elsewhere. By sequencing implementation through pilot projects, scale-up, and eventual nationwide adoption, these interventions align with the country’s evolving regulatory and data capacities. This phased design therefore embeds feasibility within the transformation framework, ensuring that proposed reforms are both context-sensitive and operationally attainable within Pakistan’s policy environment. Even so, implementing institutional reforms and digital workflows (e.g., BIM) in Pakistan will require nontrivial upfront costs, governance alignment, and long-term skills development. A follow-on study should therefore quantify feasibility and cost–benefit trade-offs, propose staged deployment pathways, and define success metrics (e.g., BIM uptake across public procurement, contractor training penetration, reductions in rework/material wastage, and third-party verification of embodied carbon reductions).

In parallel with these system-level reforms, the analysis also highlights material choice as a major driver of embodied carbon outcomes; however, the feasibility of material transitions is highly context-dependent. Steel bridge construction remains economically unfeasible at present in Pakistan due to constrained material resources, which is reflected in the absence of steel bridges along the M-13 Motorway. To benchmark the potential carbon advantages where steel is viable, an international case study of the Quincy Bayview cable-stayed bridge in the United States demonstrates that steel structures can deliver superior carbon efficiency, especially in long-span applications [63,64]. This advantage is largely attributed to steel’s high strength-to-weight ratio, its recyclability, and its compatibility with modular and prefabricated construction methods, which collectively reduce material consumption, on-site waste, and construction-phase emissions. The Quincy Bayview project illustrates how prefabricated steel systems can streamline construction while minimizing environmental impacts, underscoring the value of integrating sustainability across all stages of infrastructure delivery. Importantly, the sustainability advantages of steel, particularly its recyclability and compatibility with modular disassembly, are realized most clearly when emissions are evaluated over the full-service life rather than at construction alone. In practice, however, many infrastructure carbon studies focus on construction-stage embodied emissions because operation-stage maintenance inventories and end-of-life (EoL) recovery pathways are inconsistently reported and rarely available in comparable form. Moreover, given the long service life of major infrastructure assets (often 100 years), forecasting future maintenance regimes, rehabilitation cycles, material recovery rates, and waste-management practices with defensible accuracy is inherently difficult, even when baseline data exist. This limitation is amplified in Pakistan, where project-level maintenance records and verified recycling statistics remain scarce. As a result, the present analysis emphasizes construction-stage embodied emissions while recognizing that operational maintenance demand and EoL recovery rates can influence long-term carbon performance.

#### Implications of excluded phases

Although this study focuses on construction-stage embodied emissions due to data availability, excluding the operation and EoL phases affects the policy interpretation of the findings. During operation, emissions are mainly driven by maintenance, repair, and rehabilitation (MRR) activities (e.g., resurfacing, joint/bearing replacement, corrosion protection, and localized repairs), which depend on durability design and construction quality. Therefore, options that reduce initial embodied carbon may perform differently over the service life if they require more frequent interventions. At EoL, impacts depend on demolition practices and especially steel and concrete recovery/recycling rates; high recovery can yield meaningful benefits, whereas weak recovery systems reduce these gains. Given that M-13 emissions are dominated by concrete and reinforcement, realistic operation and EoL outcomes could change whole-life totals and potentially influence the relative performance of mitigation strategies, reinforcing the need for Pakistan-specific MRR datasets, recycling standards and infrastructure, and transparent reporting of imported material pathways.

These findings indicate a need for a coordinated sustainability strategy that extends beyond material selection. An investment in technical education to address the carbon literacy divide between stakeholders and engineers, implementation of advanced digital design tools, utilization of low-carbon materials, and regulatory reform are all critical components of this initiative. The primary focus of this study is the carbon integration that occurs during the construction phase. Nevertheless, future work should extend the framework to incorporate operation-stage MRR schedules and EoL recovery/recycling pathways as Pakistan-specific datasets become available. Furthermore, the establishment of open-access national emissions factor databases and the initiation of localized pilot projects to evaluate low-carbon materials under Pakistani construction conditions will be critical to mainstreaming sustainable infrastructure practices.

Pakistan may well be positioned to pilot approaches aligned with global sustainability; realizing this potential could require sector-wide policy adjustments. In order to achieve a balance between economic advancement and environmental accountability, the engineering and construction sectors must implement a policy transformation (from both cultural and educational perspectives) that incorporates the application of environmentally friendly materials and technologies. Pakistan has the potential to establish a resilient low-carbon infrastructure that promotes sustainable socioeconomic development and protects the environment through strategic planning, governmental support, and industrial collaboration.

## Materials and Methods

### Data collection and framework for carbon emission analysis

The M-13 Motorway, a pivotal infrastructure initiative in Pakistan, spans 117 km, linking the 2 important cities, i.e., Kharian and Rawalpindi. Developed under a PPP framework via the BOT model, this 6-lane, controlled-access highway aims to alleviate congestion on National Highway N-5 (G.T. Road), enhance regional connectivity, and stimulate economic growth by reducing travel times. As shown in Fig. S1, the motorway’s alignment incorporates critical infrastructure components such as bridges, overpass bridges, tunnels, and culverts to address challenging terrain and drainage requirements. Additional features, including interchanges, service areas, advanced safety systems, and integrated intelligent transport systems (ITS) ensure compliance with international standards while improving accessibility and operational efficiency. This study quantifies carbon emissions from constructing 52 prestressed I-girder RC structures (26 bridges and 26 overpasses) structures along the M-13 Motorway. Variations in design, dimensions, and alignment are considered to quantify the environmental impacts of their construction. Data for this analysis were collected in collaboration with the NHA and Prime Engineering and Consultant Pvt. Ltd., ensuring accuracy in material quantities, construction methods, and design specifications. However, final specifications may evolve as the project progresses. The structural details of the project are summarized in Table S5.

The data collection process was organized into 2 phases. In the first phase, essential project data were gathered, including structural design and drawings, material properties and specifications, construction techniques, and logistics and transportation details. The structural design and drawings provided crucial information on bridge types, specifications, dimensions, and structural components necessary for assessing material usage and construction methods. Material properties and specifications documented the types of materials used, their technical characteristics, and their compliance with NHA and international standards. Construction techniques and processes outlined the methods, machinery, and procedures involved, facilitating energy consumption and emissions evaluation. Additionally, logistics and transportation data covered transportation routes, material movement logistics, and adherence to NHA regulations, which were integral to assessing emissions from material transport. The material data for this study were sourced from the NHA of Pakistan and Prime Engineering and Consultant Pvt. Ltd. These data encompassed material categorization, properties, production processes, quantity and cost analysis, standards compliance, and variability in material quantities. In the second phase, data were systematically collected to identify key emission sources, such as raw material extraction and production, transportation distances, energy consumption, and machinery usage. Based on these parameters, CEFs were gathered to ensure a comprehensive emissions assessment for each bridge and overpass construction stage. Emission factors were explicitly collected for the 3 primary construction phases: raw material acquisition, transportation, and construction. All data were carefully categorized according to carbon emission metrics, life cycle stages, and bibliographic references to enhance transparency and traceability. As illustrated in Fig. S2, the database was organized systematically and geographically to ensure data reliability. Moreover, materials were classified according to their carbon emission sources, enabling a precise comparison of emission variances across different materials and regions. Material quantity data for all 52 bridges were extracted from the project’s detailed engineering drawings and bill of quantities (BOQ). For each bridge, concrete and reinforcement steel volumes/masses were compiled at the component level (deck slab, prestressed girders, diaphragms, piers, pile caps, and foundations) and then aggregated to obtain the total quantities per bridge. A sample engineering/quantity sheet used in this process is provided in the Supplementary Materials to illustrate the level of resolution and data structure. For details of the raw data, see Table S6, Data S4 and S5.

This study compiled CEFs from a variety of authoritative sources, including the IPCC Guidelines for National Greenhouse Gas Inventories, publications from the World Steel Association, regional LCA studies, and national construction databases. Additionally, data were sourced from well-established platforms such as Web of Science, Google Scholar, CNKI, Ecoinvent, and the IPCC Emission Factor Database (EFDB) (Supplementary References). The selection of these sources was guided by their credibility, with a focus on globally recognized standards and data that reflect the regional context of material production, energy mixes, and construction practices. It is important to note that the CEF of each material can vary across sources due to differences in the properties of the raw material, the production processes, regional energy mixes, data accuracy, system characteristics, unclear boundary definitions, and specific manufacturing requirements. For comprehensive CEF data, refer to Data S1 and Table S7.

### Research scope and system boundaries

The life cycle of bridges consists of 5 main phases: raw material (extraction and production), transportation, construction, services, and EoL management. Carbon emissions accumulate progressively across these phases, with the total carbon footprint calculated by aggregating the emissions from each phase. The total life cycle emissions ${\mathrm{CE}}_{\mathrm{LC}}$ can be expressed as:

$${\mathrm{CE}}_{\mathrm{LC}}={\mathrm{CE}}_{M}+{\mathrm{CE}}_{T}+{\mathrm{CE}}_{C}+{\mathrm{CE}}_{S}+{\mathrm{CE}}_{E}$$ (1)

where ${\mathrm{CE}}_{M},\;{\mathrm{CE}}_{T},\;{\mathrm{CE}}_{C},\;{\mathrm{CE}}_{S},\text{and}\;{\mathrm{CE}}_{E}$ represent the carbon emissions from raw material (extraction and production), transportation, construction, service, and EoL management, respectively. Carbon emissions from the first 3 phases, i.e., raw material (extraction and production), transportation, and construction, which are discussed in the following, are considered here in our investigations.

1. Carbon emissions from raw material (extraction and production) ${\mathrm{CE}}_{M}:$

$${\mathrm{CE}}_{M}=\sum_{i=1}^{n}Q_{i}\times {\mathrm{EF}}_{M,i}$$ (2)

where $Q_{i}\;$is the quantity of material i $,\;{\mathrm{EF}}_{M,i}$ is the emission factor for material i, and nis the total number of material types used in the first phase.

2. Carbon emissions from transportation ${\mathrm{CE}}_{T}:$

$${\mathrm{CE}}_{T}=\sum_{i=1}^{m}Q_{i}\times D_{i}\times {\mathrm{EF}}_{T,i}$$ (3)

where $Q_{i}\;$is the quantity of material i, $D_{i}$ is the transportation distance for material i, ${\mathrm{EF}}_{T,i}$ is the emission factor (measured by unit distance) for material i, and mis the total numberof material types used in the second phase.

3. Carbon emissions from construction ${\mathrm{CE}}_{C}:$

$${\mathrm{CE}}_{C}=\sum_{j=1}^{p}E_{j}\times {\mathrm{EF}}_{C,j}$$ (4)

where $E_{j}\;$is the quantity of material $j,\;{\mathrm{EF}}_{C,j}$ is the emission factor for material j, and pis the total number of material types used in this phase.

**Fig. 8.**
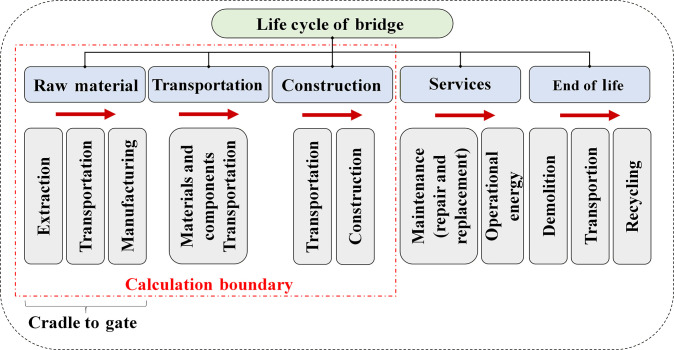
Illustration of the study boundary for the carbon emissions of bridges in the investigations. The diagram outlines the total 5 life cycle phases of bridges, with our study boundary (cradle to gate) focusing on raw material (extraction and production), transportation, and construction phases that contribute most significantly to carbon emissions.

The construction process comprises material transportation, site assembly, and other supporting operations. The operational phase primarily concerns energy consumption, replacement tasks, and service maintenance. Recycling, waste transportation, and demolition are indispensable operations during the EoL phase. Figure 8 illustrates the system boundaries established for the investigation, which consist of 3 primary phases: construction, transportation, and raw material (extraction and production).

**Fig. 9.**
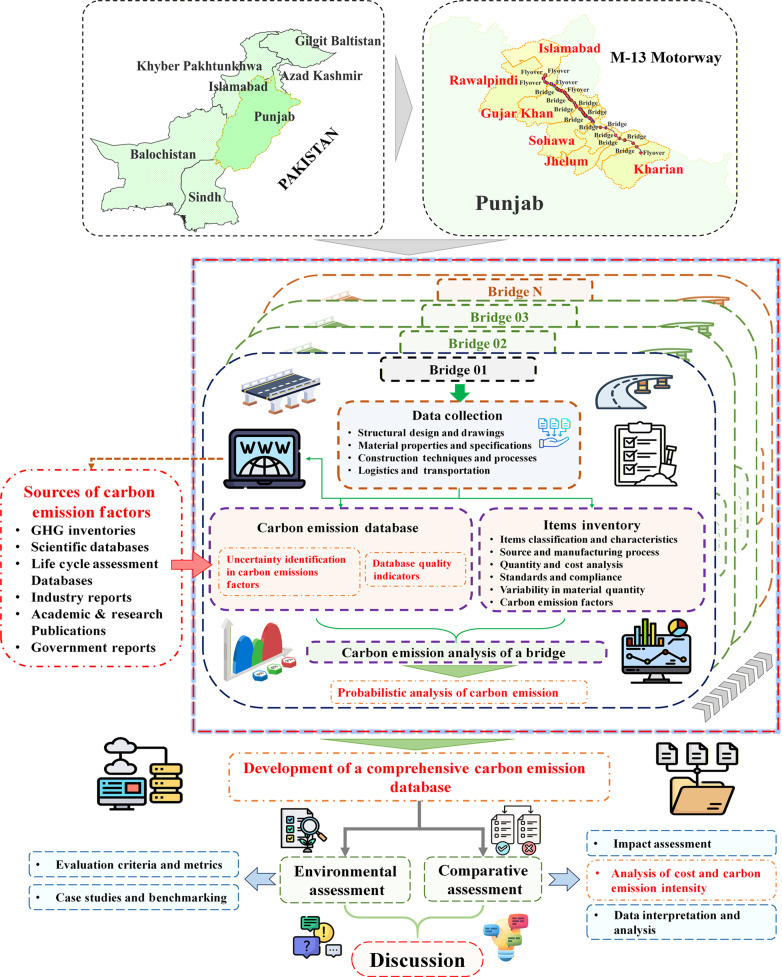
Framework for carbon emission analysis of bridge construction. The process includes identification of carbon emission sources, creation of a structured emission factor database, and execution of the probabilistic analyses. It integrates environmental and comparative assessments, item inventories, and database quality checks, ultimately leading to informed discussion through impact assessment and benchmarking.

The investigations of the present paper concentrate on the 3 critical phases of raw material (extraction and production), transportation, and construction due to their substantial contribution to carbon emissions in bridge construction. These phases are typically responsible for most emissions, especially when high-carbon materials such as steel and concrete are involved. The operation and EoL phases were excluded from the analysis, as they generally have relatively lower emissions and are more challenging to estimate accurately, owing to the lack of long-term reliable data on quantities and inconsistencies in maintenance and recycling practices. Data for the first 3 phases were relatively accessible and reliable, aligning with our study's focus on embodied carbon. Our study employed probabilistic approaches to assess the uncertainty in carbon emissions during bridge construction, specifically MCSs, which allows for the evaluation of parameter uncertainty, helping to pinpoint key processes that contribute to total emissions and overall uncertainty. Particular attention was paid to the coefficients of variation (CVs) to quantify the variability in the emission estimation. The methodological framework used for this analysis is illustrated in Fig. 9. Although the framework was developed for Pakistan’s bridge infrastructure, its methodological structure is broadly adaptable to other regions and infrastructure types. By integrating probabilistic MCSs with emission factor uncertainty evaluation, functional normalization, and policy linkage, the approach can be replicated under varying data conditions and design codes. Because its parameters can be recalibrated using local emission factors, cost data, or standards, the framework can readily extend to tunnels, highways, and other transportation systems in different contexts.

### CEF evaluation with uncertainty identification

Accurately determining CEFs for bridge construction materials is crucial for assessing the environmental impact of infrastructure projects. However, uncertainties significantly affect these assessments. Variability in manufacturing processes, including differences in production technologies and operational efficiencies, leads to substantial variations in CEFs. For instance, the carbon intensity of steel production can fluctuate significantly depending on furnace types, feedstock sources, and recycling practices. Ambiguities in the origins of raw materials further complicate the accurate assessment of their true carbon footprints, as geographically dispersed supply chains often result in heterogeneous carbon profiles. Additionally, inconsistencies in testing protocols and measurement techniques across different studies create comparative challenges. Different studies might employ varying standards or system boundaries, making direct comparisons difficult [55,65,66]. Moreover, uncertainties inherent in LCAs stem from differing definitions of system boundaries, such as inclusion or exclusion of transportation processes, manufacturing stages, and EoL disposal procedures, which can significantly alter estimated emission factors.

Temporal variability presents additional complexity; advancements in production methods, shifts in regulatory frameworks, and improvements in energy efficiency result in continuous changes in carbon intensities over time. Such temporal shifts are particularly pronounced in the energy-intensive production of materials like cement and steel, where innovation and regulatory policies frequently alter emission profiles. Furthermore, reliance on generic data or proxy values due to data gaps can introduce significant errors, and measurement inaccuracies from primary data collection processes compound this uncertainty. Energy grid variability also contributes substantially to uncertainty, as regional differences in electricity generation mixes ranging from fossil-fuel-dominated to renewable energy sources result in markedly different carbon intensities for electricity consumed during material production. The resultant uncertainty in emission factors associated with energy-dependent processes underscores the importance of regional specificity in carbon assessments [67,68].

To systematically manage these uncertainties, a comprehensive probabilistic analysis was performed, assigning statistical distributions to each identified source of uncertainty. Such detailed uncertainty quantification is essential to inform policymakers and practitioners, facilitating targeted interventions to mitigate GHG emissions effectively in infrastructure projects.

### Probabilistic analysis of carbon emissions of large-scale bridges

The probabilistic analysis of carbon emissions for large-scale bridges is crucial for understanding the uncertainties associated with infrastructure projects. Several classification frameworks have been proposed in the literature to categorize these uncertainties. Notably, PAS 2050 distinguishes between uncertainties stemming from technical factors (e.g., data collection methods and modeling assumptions) and natural variability (e.g., inherent fluctuations in material properties or environmental conditions) [69]. Similarly, the classification framework proposed by Tian et al. [70] differentiates between aleatory uncertainty (inherent randomness in systems) and epistemic uncertainty (resulting from limited knowledge or data). Through an extensive literature review, He et al. [71] elaborated on these distinctions per PAS 2050:2011, providing a detailed analysis of how technical and natural variability uncertainties manifest in carbon footprint assessments. Other studies have contributed to this discourse by introducing additional dimensions of uncertainty. For instance, Lloyd and Ries [67] proposed a framework that categorizes uncertainties into parameters, models, and scenarios, emphasizing the need for tailored approaches to address each type. Meanwhile, Mutel and Hellweg [72] highlighted the role of spatial and temporal variability as key sources of uncertainty in LCA, particularly for large-scale infrastructure projects. Additionally, Lueddeckens et al. [73] introduced the concept of decision-driven uncertainty, which arises from the subjective choices made during the LCA process, such as system boundary selection and functional unit definition. Recent probabilistic digital twin frameworks further extend this perspective by integrating uncertainty propagation directly into construction-phase carbon modeling, enabling dynamic, data-driven refinement of emission estimates [74].

This study primarily focuses on parametric uncertainty, which arises from the variability in input data elements particularly CEFs, due to inconsistencies in datasets and existing knowledge gaps. Probabilistic methods were employed to address these uncertainties. Specifically, MCS was utilized as a stochastic analysis tool, generating data samples based on predefined input distributions. The accuracy of carbon emission assessments is heavily influenced by variations in the LCI data, with CEFs being a key source of uncertainty. Both probabilistic and semiquantitative approaches were adopted to investigate parameter uncertainty thoroughly. However, defining appropriate probabilistic distributions and statistical parameters typically requires extensive datasets, which are often unavailable for bridge construction projects due to the diverse range of data sources and their limited availability. Accordingly, uncertainty is propagated by treating key input parameters as random variables, primarily the material CEFs and the material quantities used in the inventory. In the Pakistani context, CEFs are epistemically uncertain because locally verified environmental product declaration (EPD)/LCI datasets are scarce and available emission factors are compiled from heterogeneous sources with differing temporal/geographical representativeness and system boundary assumptions. In addition, material quantities can exceed nominal take-off values due to unavoidable construction wastage and losses during transportation, storage, handling, and installation; therefore, quantities were conservatively modeled with a 0% to +10% allowance relative to nominal values to represent these expected losses.

To address the challenges posed by data uncertainty, this study employs a DQI-based approach, which assesses data quality based on several descriptive metadata categories, including source reliability, temporal relevance, geographical correlation, and technological consistency. These qualitative assessments are then converted into probabilistic distributions through expert judgment and empirical transformation relationships. Previous research emphasizes 5 key factors in environmental assessment data evaluation: source reliability, data completeness, temporal relevance, geographical correlation, and technological consistency [75–77]. These indicators are particularly relevant for infrastructure projects, where emission factors and material data may vary significantly due to differences in production methods, regional practices, and temporal changes. For example, emission factors for materials like concrete and steel can differ based on the energy sources used in production or the geographical location of manufacturing facilities [78,79]. This framework differentiates between fundamental uncertainty (stemming from inherent ambiguities within datasets) and additional uncertainty (arising from estimation errors and data variability). The DQI-based method relies on several key assumptions: (a) independence of indicators, where DQIs are treated as independent variables, and (b) transformation to probabilistic distributions, where the grades of quality indicators are converted into probability distributions using empirical relationships.

A semiquantitative framework was used to address parameter uncertainty in carbon emission assessments using DQIs. A 7 × 5 matrix was developed to systematically evaluate 7 criteria: source, reliability, temporal correlation, geographical correlation, data consistency, adequacy of references, and data completeness. Technological consistency was excluded due to the lack of detailed information on the production process across diverse data sources. Each criterion was graded on a 5-level scale (1 = low quality, 5 = optimal quality). Higher values of $Q_{i}$correspond to recent, regionally relevant, well-documented data with consistent units and minimal gaps, while lower values indicate older, nonregional, or poorly documented sources with missing values, enabling standardized emission factor data quality scoring. See Data S6 to S8 for DQI criteria, scoring rule, and details.

The SDs derived separately from the DQI scores $\sigma_{\mathrm{DQI}}$ and CEFs $\sigma_{\mathrm{CF}}\;$were calculated using Eqs. (5) and (6), respectively:

$$\sigma_{\mathrm{CF}}=\sqrt{\frac{1}{n}\sum_{i}^{n}{\left[x_{i}-\mu \right]}^{2}}$$ (5)

$$\sigma_{\mathrm{DQI}}=\sqrt{\frac{1}{n}\sum_{i}^{n}{\left[{\mathrm{DQI}}_{i}-\mu_{\mathrm{DQI}}\right]}^{2}}$$ (6)

We combine the uncertainties identified above through the error propagation approach, resulting in a comprehensive combined uncertainty $\sigma_{\text{Combined}}$ as expressed in Eq. (7):

$$\sigma_{\text{Combined}}=\sqrt{\sigma_{\mathrm{CF}}^{2}+\sigma_{\mathrm{DQI}}^{2}}$$ (7)

In the probabilistic model, the DQI contribution is operationalized as an additional dispersion term $\sigma_{\mathrm{DQI}}$​, which are combined with the dataset-derived emission factor dispersion $\sigma_{\mathrm{CF}}$​ via Eq. (7) to obtain $\sigma_{\text{Combined}}$​. These DQI-adjusted parameters define the spread of CEF inputs used for subsequent distribution-fitting and Monte Carlo sampling. These combined uncertainties facilitated the adjustment of both the mean and the SD values for the CEFs, thus providing adjusted statistical parameters for further probabilistic analysis. The adjusted mean and adjusted SD values were subsequently employed as the key parameters in selecting appropriate probability distributions. Candidate distributions (e.g., normal, log-normal, gamma, and Weibull) were fitted by maximum likelihood estimation and then subjected to rigorous goodness-of-fit testing. The Anderson–Darling (AD) test [80] and the Kolmogorov–Smirnov (KS) test [81] were utilized to evaluate the adherence of emission factors to a particular distribution, which is essential for establishing the reliability of the probabilistic model. The formula for the AD test statistic ($A^{2}$) is:

$$A^{2}=-n\sum_{i=1}^{n}\frac{\left(2i-1\right)}{n}\left[\text{In}\left(F\left(x_{i}\right)\right)+\text{In}\left(1-F\left(x_{n+1-i}\right)\right)\right]$$ (8)

where $F\left(x_{i}\right)$ represents the cumulative distribution function for the hypothesized distribution and $x_{i}\;$is the sorted sample data. Similarly, the KS test statistic D is computed as:

$$D=\max_{i}|F_{n}\left(x_{i}\right)-F\left(x_{i}\right)|$$ (9)

where $F_{n}\left(x_{i}\right)$ is the empirical cumulative distribution function (CDF) of the sample and $F\left(x_{i}\right)$ is the CDF of the reference distribution. Distributions were accepted only if both AD and KS tests yielded P>0.05 and if the Akaike information criterion (AIC) was at its minimum

$$\mathrm{AIC}=2k-2\text{In}\left(L\right)$$ (10)

where k is the number of estimated parameters in the model and L is the likelihood of the model. Distributions were selected based on the lowest AIC value, and in cases where ΔAIC<2 between 2 viable candidates, both were retained for downstream sensitivity analysis. While this approach may not match the precision of purely statistical methods, it provided a cost-effective and holistic assessment of uncertainty, which is particularly critical for data-scarce bridge construction projects. Detailed fit statistics (AD, KS, and AIC) for each material are provided in Data S9. The materials concrete D2, concrete A1, reinforcement ASTM-A-36, and Diesel are the primary contributors to carbon emissions and the main sources of significant variability in total emissions. Their quantile–quantile (Q–Q) plots and convergence of Monte Carlo statistics for the emission factor datasets are presented in Figs. S3 and S4. Other materials exhibit similar patterns, demonstrating the agreement between the empirical data and the fitted normal distributions. The DQI ratings, as shown in Fig. S5, highlight the variability in data quality across the criteria and emphasize the importance of using a structured framework to ensure reliable emission assessments. A comprehensive discussion of the methodology is available in [82].

Finally, the adjusted distribution parameters and selected distribution families were integrated into a Monte Carlo framework. Across 10,000 iterations, randomized emission factor samples generated project-level emission realizations to propagate parameter uncertainty; convergence was tracked via the stability of the mean and the 5th/95th percentiles as run length increased, and *N* = 10,000 satisfied our prespecified tolerance. Unless otherwise noted, input draws were treated as independent; practical dependencies (e.g., co-movement among item quantities within a bridge or phase) were handled within the inventory quantities rather than by correlating emission factor samples. Sensitivity was evaluated through one-at-a-time perturbations and variance attribution at the phase/material level, which consistently identified reinforcement and concrete as dominant contributors. The simulations produce probability distributions for total emissions that reflect the full spectrum of plausible outcomes; Fig. S6 illustrates how CEFs and DQIs are combined to yield comprehensive, realistic emission distributions.

### Transportation infrastructure carbon emission indicator

To align with global sustainability objectives and enhance environmental performance, it is essential to assess the carbon efficiency of bridges and other transportation infrastructures. To further support effective benchmarking, we hereby define one indicator, i.e., CEI, for bridges and other transportation infrastructures. CEI quantifies the carbon emissions produced per unit length of bridges, roads, and tunnels. The standardized formula for CEI is expressed as a function of the policies p and the transportation length from a probablistic perspective:

$$C{\mathrm{EI}}_{A}\left(p;L,B\right)=\frac{E\left[\text{Total}\;\text{Carbon Emission}\;\left(p\right)\right]}{L\times B}$$ (11)

where $E\left[\cdot \right]$ denotes the expected value of total emissions given the policies, p capturing variability in materials, energy sources, and construction processes. In cases of skewed emission distributions, the median or selected percentiles may be reported alongside the mean to convey uncertainty comprehensively. The denominators L and *B* are treated as the nominal functional output (i.e., bridge length and deck width) and treated as deterministic based on design tolerances. This definition aligns with international product carbon footprint standards such as ISO 14067 and related life cycle assessment guidelines, facilitating policy-sensitive comparisons across diverse infrastructure types.

This indicator enables fair quantitatively comparisons across the transportation infrastructures by systematically accounting for emissions, thus informing targeted policy transformation for improved sustainability and environmental performance. The proposal of this indicator can further support the selection of the infrastructures (e.g., the selection of the bridge type or the selection of the transportation mode) efficiently based on the comparison across diverse types of bridges and other potential appropriate transportation infrastructures. However, even when CEI is normalized by deck area, structural and geotechnical differences remain across infrastructure classes. Tunnels involve substantial excavation and lining, while bridges differ in span arrangement, pier height, and foundation systems, all of which influence material intensity and embodied carbon. In this study, CEI comparisons are therefore interpreted as indicative rather than strictly like-for-like, with particular emphasis placed on RC highway bridges that are typologically closest to the M-13 corridor.

## Data Availability

All the public data used in this research have been cited and described within the article. Any additional datasets or processed data generated during the current study are available from the corresponding author upon reasonable request, and no materials subject to a material transfer agreement were used. Code is available upon request.

## References

[1] Khahro SH, Kumar D, Siddiqui FH, Ali TH, Raza MS, Khoso AR. Optimizing energy use, cost and carbon emission through building information modelling and a sustainability approach: A case-study of a hospital building. Sustainability. 2021;13(7):3675.

[2] Passer A, Lutzkendorf T, Habert G, Kromp-Kolb H, Monsberger M, Eder M, Truger B. Sustainable built environment: Transition towards a net zero carbon built environment. Int J Life Cycle Assess. 2020;25(6):1160–1167.

[3] Miller SA, Moore FC. Climate and health damages from global concrete production. Nat Clim Chang. 2020;10(5):439–443.

[4] Labaran YH, Mathur VS, Muhammad SU, Musa AA. Carbon footprint management: A review of construction industry. Clean Eng Technol. 2022;9: Article 100531.

[5] Tian F, Pang Z, Hu S, Zhang X, Wang F, Nie W, Xia X, Li G, Hsu H-Y, Xu Q, et al. Recent advances in electrochemical-based silicon production technologies with reduced carbon emission. Research. 2023;6:0142. 37214200 10.34133/research.0142 PMC10194053

[6] Sun Y, Wen G, Dai H, Feng Y, Azaele S, Lin W, Zhou F. Quantifying the resilience of coal energy supply in China toward carbon neutrality. Research. 2024;7:0398. 39015205 10.34133/research.0398 PMC11249919

[7] Zhao Y, Ma J, Li Y, Cheng K, Zhang M, Liu Z, Yang F. Carbon emission based predictions of anthropogenic impacts on groundwater storage at typical basins in 2050. Research. 2025;8:0680. 40458612 10.34133/research.0680 PMC12129122

[8] Rydge J, Jacobs M, Granoff I. Ensuring new infrastructure is climate-smart. In: Contributing paper for seizing the global opportunity: Partnerships for better growth and a better climate. London and Washington (DC): New Climate Economy; 2015.

[9] Gillingham KT, Huang C, Buehler JP, Gentner DR. The climate and health benefits from intensive building energy efficiency improvements. Sci Adv. 2021;7(34):eabg0947. 34417173 10.1126/sciadv.abg0947 PMC8378816

[10] Khalid HH, Li Z, He H, Hazib HM, Peng Z. Toward clean air in developing countries: A comparative analysis of PM2.5 trajectories under emission-reduction policies in China and India. *Atmos Pollut Res*. 2026:102939.

[11] Lenton TM, Xu C, Abrams JF, Ghadiali A, Loraini S, Sakschewski B, Zimm C, Ebi KL, Dun RR, Svenning J-C. Quantifying the human cost of global warming. Nat Sustainability. 2023;6(10):1237–1247.

[12] Cabernard L, Pfister S, Oberschelp C, Hellweg S. Growing environmental footprint of plastics driven by coal combustion. Nat Sustainability. 2022;5(2):139–148.

[13] Pervez H, Ali Y, Petrillo A. A quantitative assessment of greenhouse gas (GHG) emissions from conventional and modular construction: A case of developing country. TERI Info Digest Energy Environ. 2022;21(2):219–220.

[14] Bruckner B, Hubacek K, Shan Y, Zhong H, Feng K. Impacts of poverty alleviation on national and global carbon emissions. Nat Sustainability. 2022;5(4):311–320.

[15] Buffenbarger JK, Casilio JM, AzariJafari H, Szoke SS. Role of mixture overdesign in the sustainability of concrete: Current state and future perspective. ACI Mater J. 2023;120(1):89–100.

[16] Budd O, Hawkins W. Trimming the structural ‘fat’: The carbon cost of overdesign in bridges. Int fib Symp Concept Des Struct. 2021;2021:375–382.

[17] Contestabile M. Narratives for change. Nat Sustainability. 2021;4(12):1017.

[18] Novokshchenov V. Computer-aided statistical evaluation of the strength data obtained during construction of Khairabad bridge in Pakistan. Mater Struct. 1999;32(6):452–459.

[19] Ahmad R, Liu G, Rehman SAU, Fazal R, Gao Y, Xu D, Agostinho F, Almeida CMVB, Giannetti BF. Pakistan road towards Paris Agreement: Potential decarbonization pathways and future emissions reduction by a developing country. Energy. 2025;314: Article 134075.

[20] García-Segura T, Penadés-Plà V, Yepes V. Sustainable bridge design by metamodel-assisted multi-objective optimization and decision-making under uncertainty. J Clean Prod. 2018;202:904–915.

[21] Liu Y, Wang Y, Li D. Estimation and uncertainty analysis on carbon dioxide emissions from construction phase of real highway projects in China. J Clean Prod. 2017;144:337–346.

[22] Nagendra H, Bai X, Brondizio ES, Lwasa S. The urban south and the predicament of global sustainability. Nat Sustainability. 2018;1(7):341–349.

[23] Yousuf I, Ghumman A, Hashmi HN, Kamal MA. Carbon emissions from power sector in Pakistan and opportunities to mitigate those. Renew Sust Energ Rev. 2014;34:71–77.

[24] Rehman A, Ma H, Ozturk I. Do industrialization, energy importations, and economic progress influence carbon emission in Pakistan. Environ Sci Pollut Res. 2021;28(33):45840–45852. 10.1007/s11356-021-13916-4 33881694

[25] Huang YA, Weber CL, Matthews HS. Categorization of scope 3 emissions for streamlined enterprise carbon footprinting. Environ Sci Technol. 2009;43(22):8509–8515. 20028044 10.1021/es901643a

[26] Peng C. Calculation of a building's life cycle carbon emissions based on Ecotect and building information modeling. J Clean Prod. 2016;112:453–465.

[27] Onat NC, Kucukvar M, Tatari O. Scope-based carbon footprint analysis of US residential and commercial buildings: An input–output hybrid life cycle assessment approach. Build Environ. 2014;72:53–62.

[28] Lenzen M. Errors in conventional and input-output–based life–cycle inventories. J Ind Ecol. 2000;4(4):127–148.

[29] van Greevenbroek K, Grochowicz A, Zeyringer M, Benth FE. Trading off regional and overall energy system design flexibility in the net-zero transition. Nat Sustain. 2025;8.:629–641.

[30] Suh S, Huppes G. Methods for life cycle inventory of a product. J Clean Prod. 2005;13(7):687–697.

[31] Bertassoli DJ Jr, Sawakuchi HO, de Araujo KR, de Camargo MGP, Alem VAT, Pepeira TS, Krusche AV, Batviken D, Richey JE, Sawakuchi AO, How green can Amazon hydropower be? Net carbon emission from the largest hydropower plant in Amazonia. Sci Adv. 2021;7(26):eabe1470. 34172455 10.1126/sciadv.abe1470 PMC8232918

[32] Hazib HM, Shabbir I, Lai W, Pan Y, Qin J. Uncertainty propagation in bridge construction carbon emission quantification. In: *Proceedings of the 15th International Conference on Quality, Reliability, Risk, Maintenance, and Safety Engineering & 8th International Conference on Materials and Reliability*. 2025. p. 1–6.

[33] Nasir S, Kucerik J, Mahmood Z. A study on the washability of the Azad Kashmir (Pakistan) coalfield. Fuel Process Technol. 2012;99:75–81.

[34] Mahjoubi S, AzariJafari H, Kirchain R, Olivetti E. Assessing the impact of overdesign on concrete embodied emissions. Cambridge (MA: MIT Concrete Sustainability Hub; 2025.

[35] Du G, Safi M, Pettersson L, Karoumi R. Life cycle assessment as a decision support tool for bridge procurement: Environmental impact comparison among five bridge designs. Int J Life Cycle Assess. 2014;19(12):1948–1964.

[36] Kaewunruen S, Sresakoolchai J, Zhou Z. Sustainability-based lifecycle management for bridge infrastructure using 6D BIM. Sustainability. 2020;12(6):2436.

[37] Liu T, Zhu H, Shen Y, Li T, Liu A. Embodied carbon assessment on road tunnels using integrated digital model: Methodology and case-study insights. Tunn Undergr Space Technol. 2024;143: Article 105485.

[38] Adim L. *Planning law assessment framework*. Nairobi (Kenya): UN-Habitat (United Nations Human Settlements Programme); 2018.

[39] Allwood JM, Gutowski TG, Serrenho AC, Skelton AC, Worrell E. Industry 1.61803: The transition to an industry with reduced material demand fit for a low carbon future. Philos Trans A Math Phys Eng Sci. 2017;375(2095): Article 20160361. 28461426 10.1098/rsta.2016.0361 PMC5415643

[40] Shah A, Qureshi M, Saleem W, Naseer S, Haq I-U. An analysis of seismic provisions of building code of Pakistan. In: *5th World Engineering Congress (WEC 2013)*. Islamabad (Pakistan): National University of Sciences & Technology; 2013. p. 23–25.

[41] Rehman AU, Bashir MT, Khan MMH, Qureshi HA, Uddin MA, Ahmed K. Calibration of load and resistance factor rating for short to medium-span lengths bridges in Pakistan. Iran J Sci Technol Trans Civil Eng. 2025.

[42] Dawn, *Building standards: Fit for purpose?* Karachi (Sindh, Pakistan): Dawn; 2024.

[43] Burney J, Persad G, Proctor J, Bendavid E, Burke M, Heft-Neal S. Geographically resolved social cost of anthropogenic emissions accounting for both direct and climate-mediated effects. Sci Adv. 2022;8(38):eabn7307. 36149961 10.1126/sciadv.abn7307 PMC9506714

[44] Climate Transparency. Pakistan country profile 2020. Climate Transparency, 2021. https://www.climate-transparency.org/wp-content/uploads/2021/11/Pakistan-CP-2020.pdf.

[45] EU SWITCH-Asia Sustainable Consumption, Facility, Pakistan Ministry of Climate Change. Vision 2030 for a Green Building Code in Pakistan. EU SWITCH-Asia SCP Facility, 2022. https://www.switch-asia.eu/site/assets/files/3366/pakistan_vision_2030_final.pdf.

[46] World Green Building Council. Pakistan’s another step towards the decarbonisation of its built environment and its socio-economic impact. https://worldgbc.org/article/pakistans-another-step-towards-the-decarbonisation-of-its-built-environment-and-its-socio-economic-impact/.

[47] Meckling J, Karplus VJ. Political strategies for climate and environmental solutions. Nat Sustainability. 2023;6(7):742–751.

[48] Grassi G, Peters GP, Canadell JG, Cescatti A, Federici S, Gidden MJ, Harris N, Herold M, Krug T, O’Sullivan M, et al. Improving land-use emission estimates under the Paris Agreement. Nat Sustain. 2025;8:579–581.

[49] Crossrail Ltd. *Sustainability summary 2018*. *Crossrail Learning Legacy*. London (UK): Crossrail Ltd.; 2018. https://learninglegacy.crossrail.co.uk/wp-content/uploads/2018/07/Sustainability-Summary-2018.pdf.

[50] The University of Manchester. How can we make concrete greener (and cheaper)? https://www.manchester.ac.uk/research/structure/platforms/sustainable-futures/climate-questions/mitigation/concretene/.

[51] Shpak N, Kyrylych T, Greblikaitė J. Diversification models of sales activity for steady development of an enterprise. Sustainability. 2016;8(4):393.

[52] Mountaki D. *Computational optimization of hempcrete integration: Improving energy performance and minimizing embodied energy in a variety of building types and climates*. Delft (Netherlands): Delft University of Technology; 2024.

[53] El Husseini F, Noura HN, Salman O, Chahine K. Machine learning in smart buildings: A review of methods, challenges, and future trends. Appl Sci. 2025;15(14):7682.

[54] Lai W, Hu R, Qin J, Pan Y, Chen J. Digital twin-based quantification of carbon emission from foundation pit construction: A case study of a station in Shanghai Metro. IOP Conf Ser Earth Environ Sci. 2024;1337:012049.

[55] Chen C, Kirabaeva K, Massetti E, Minnett D, Parry I, Tim T, von Thadden-Kostopoulos S, Eur GD. Assessing recent climate policy initiatives in the Netherlands Washington (DC): International Monetary Fund; 2023.

[56] Wouters K, de Simon L. Kwantitatieve Evaluatie Green Deal Duurzaam GWW 2.0. TNO–Netherlands Organisation for Applied Scientific Research, Utrecht, 2021-01-08 2021. https://publications.tno.nl/publication/34638002/lPHY8A/TNO-2020-R12296.pdf

[57] Li X, Wang C, Kassem MA, Wu S-Y, Wei T-B. Case study on carbon footprint life-cycle assessment for construction delivery stage in China. Sustainability. 2022;14(9):5180.

[58] Manifold J, Renukappa S, Suresh S, Georgakis P, Perera GR. Dual transition of net zero carbon and digital transformation: Case study of UK transportation sector. Sustainability. 2024;16(17):7852.

[59] High Speed Two (HS2) Limited. Environmental sustainability progress report 2024–2025. High Speed Two (HS2) Limited, Birmingham; 2025-08-12 2025. https://assets.hs2.org.uk/wp-content/uploads/2025/07/BC0071_HS2_A4_Lscape_48pg_ESPR-2025_V3_FINAL31.pdf

[60] Beck R, Spasovski J, Gentile L, Mukkamala AM. *EmisChain—An application of blockchain technology for road-transport emissions monitoring to support an emissions market and an in-use emissions-based tolling system in the EU*. Copenhagen (Denmark): IT-University of Copenhagen; 2019.

[61] Alaux N, Saade MRM, Passer A. Inventory regionalization of background data: Influence on building life cycle assessment and carbon reduction strategies. J Clean Prod. 2024;459: Article 142434.

[62] Vaz I, Scolaro T, Schaefer A, Thives L, Ghisi E. A comparison of local and global databases for the environmental impact of residential buildings. In: *Proceedings of The World Conference on Climate Change and Global Warming*. Sisimiut (Greenland): Diamond Scientific Publishing; 2025.

[63] Franchini A, Galasso C. Seismic optimisation of cable-stayed bridges based on expected annual loss and embodied carbon. In: *SECED 2023 Conference-Earthquake Engineering and Dynamics for a Sustainable Future*. Cambridge (UK): Society for Earthquake and Civil Engineering Dynamics (SECED); 2023.

[64] Wilson JC, Gravelle W. Modelling of a cable-stayed bridge for dynamic analysis. Earthq Eng Struct Dyn. 1991;20(8):707–721.

[65] Richardson S, Hyde K, Connaughton J. Uncertainty assessment of comparative design stage embodied carbon assessments. In: Embodied carbon in buildings: Measurement, management and mitigation. Cham (Switzerland): Springer International Publishing; 2018. p. 51–76.

[66] Bawden KR, Williams ED, Babbitt CW. Mapping product knowledge to life cycle inventory bounds: A case study of steel manufacturing. J Clean Prod. 2016;113:557–564.

[67] Lloyd SM, Ries R. Characterizing, propagating, and analyzing uncertainty in life-cycle assessment: A survey of quantitative approaches. J Ind Ecol. 2007;11(1):161–179.

[68] Zhai B. *Carbon emission reduction benefits analysis of recycled fill materials in subway station construction waste for roadbed backfilling*. Zhengzhou (People’s Republic of China): North China University of Water Resources and Electric Power; 2023.

[69] Pomponi F, Moncaster A. Scrutinising embodied carbon in buildings: The next performance gap made manifest. Renew Sust Energ Rev. 2018;81:2431–2442.

[70] Tian W, Heo Y, De Wilde P, Li Z, Yan D, Park CS, Feng X, Augenbroe G. A review of uncertainty analysis in building energy assessment. Renew Sust Energ Rev. 2018;93:285–301.

[71] He B, Pan Q, Deng Z. Product carbon footprint for product life cycle under uncertainty. J Clean Prod. 2018;187:459–472.

[72] Mutel CL, Hellweg S. Regionalized life cycle assessment: Computational methodology and application to inventory databases. Environ Sci Technol. 2009;43(15):5797–5803. 19731679 10.1021/es803002j

[73] Lueddeckens S, Saling P, Guenther E. Temporal issues in life cycle assessment—A systematic review. Int J Life Cycle Assess. 2020;25(8):1385–1401.

[74] Lai W, Pan Y, Zhang L, Chen J-J, Qin J. Towards low-carbon construction of metro station foundation pit: A probabilistic digital twin framework with self-supervised learning capability. *Tunn Undergr Space Technol*. 2026;171:107450.

[75] May JR, Brennan DJ. Application of data quality assessment methods to an LCA of electricity generation. Int J Life Cycle Assess. 2003;8(4):215–225.

[76] Hong J, Shen GQ, Peng Y, Feng Y, Mao C. Uncertainty analysis for measuring greenhouse gas emissions in the building construction phase: A case study in China. J Clean Prod. 2016;129:183–195.

[77] Wang E, Shen Z. A hybrid data quality indicator and statistical method for improving uncertainty analysis in LCA of complex system—Application to the whole-building embodied energy analysis. J Clean Prod. 2013;43:166–173.

[78] Zhang X, Liu K, Zhang Z. Life cycle carbon emissions of two residential buildings in China: Comparison and uncertainty analysis of different assessment methods. J Clean Prod. 2020;266: Article 122037.

[79] Weidema BP, Bauer C, Hischier R, Mutel C, Nemecek T, Reinhard J, Vadenbo CO, Wernet G. *Overview and methodology: Data quality guideline for the ecoinvent database version 3*. St. Gallen (Switzerland): The Ecoinvent Centre Publisher; 2013.

[80] Scholz FW, Stephens MA. *K*-sample Anderson–Darling tests. J Am Stat Assoc. 1987;82(399):918–924.

[81] Massey FJ Jr. The Kolmogorov-Smirnov test for goodness of fit. J Am Stat Assoc. 1951;46(253):68–78.

[82] Zhang X, Wang F. Stochastic analysis of embodied emissions of building construction: A comparative case study in China. Energ Buildings. 2017;151:574–584.

[83] Ritchie H, Rosado P, Roser M. CO₂ and greenhouse gas emissions. In: Our world in data. Oxford (UK): Global Change Data Lab; 2023.

