# Supplementary material for: Probabilistic Carbon Analysis of Pakistan’s Bridges Unveils the Urgent Needs of Overdesign Optimization and Policy Transformation
Source: Research (Wash D C). 2026 Mar 3;7:1175. doi: 10.34133/research.1175 (PMC12953927; doi:10.34133/research.1175)
Supplement: Supplementary 1 — Figs. S1 to S6 Data S1 to S9 [file research.1175.f1.zip › Fig. S3.pdf]

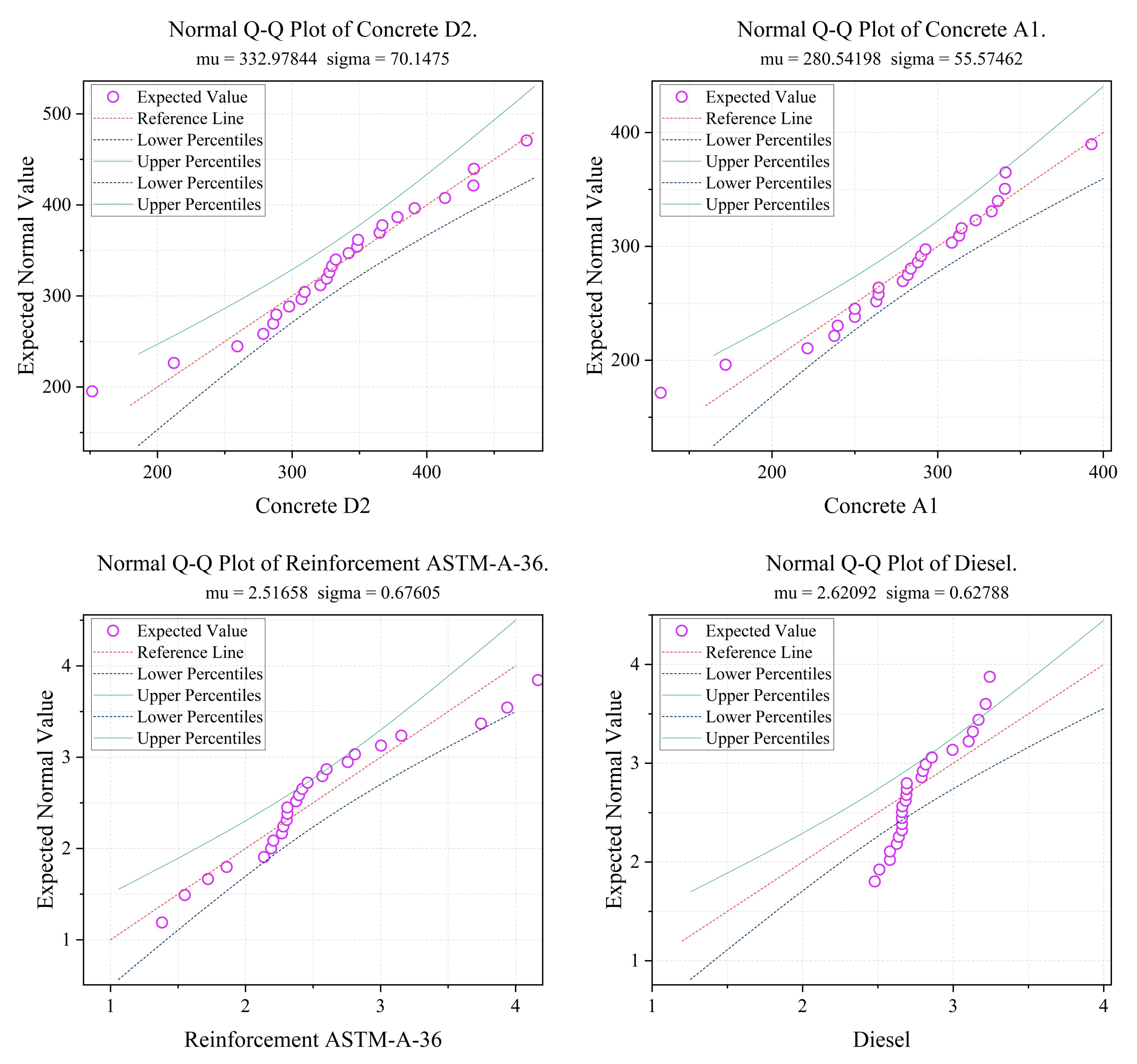

Normal Q-Q Plot of Concrete D2.
mu = 332.97844 sigma = 70.1475
Expected Value
Reference Line
Lower Percentiles
Upper Percentiles
Lower Percentiles
Upper Percentiles
Expected Normal Value
Concrete D2
Normal Q-Q Plot of Concrete A1.
mu = 280.54198 sigma = 55.57462
Expected Value
Reference Line
Lower Percentiles
Upper Percentiles
Lower Percentiles
Upper Percentiles
Expected Normal Value
Concrete A1
Normal Q-Q Plot of Reinforcement ASTM-A-36.
mu = 2.51658 sigma = 0.67605
Expected Value
Reference Line
Lower Percentiles
Upper Percentiles
Lower Percentiles
Upper Percentiles
Expected Normal Value
Reinforcement ASTM-A-36
Normal Q-Q Plot of Diesel.
mu = 2.62092 sigma = 0.62788
Expected Value
Reference Line
Lower Percentiles
Upper Percentiles
Lower Percentiles
Upper Percentiles
Expected Normal Value
Diesel
