# Supplementary figures and images for: Probabilistic Carbon Analysis of Pakistan’s Bridges Unveils the Urgent Needs of Overdesign Optimization and Policy Transformation
Source: Research (Wash D C). 2026 Mar 3;7:1175. doi: 10.34133/research.1175 (PMC12953927; doi:10.34133/research.1175)

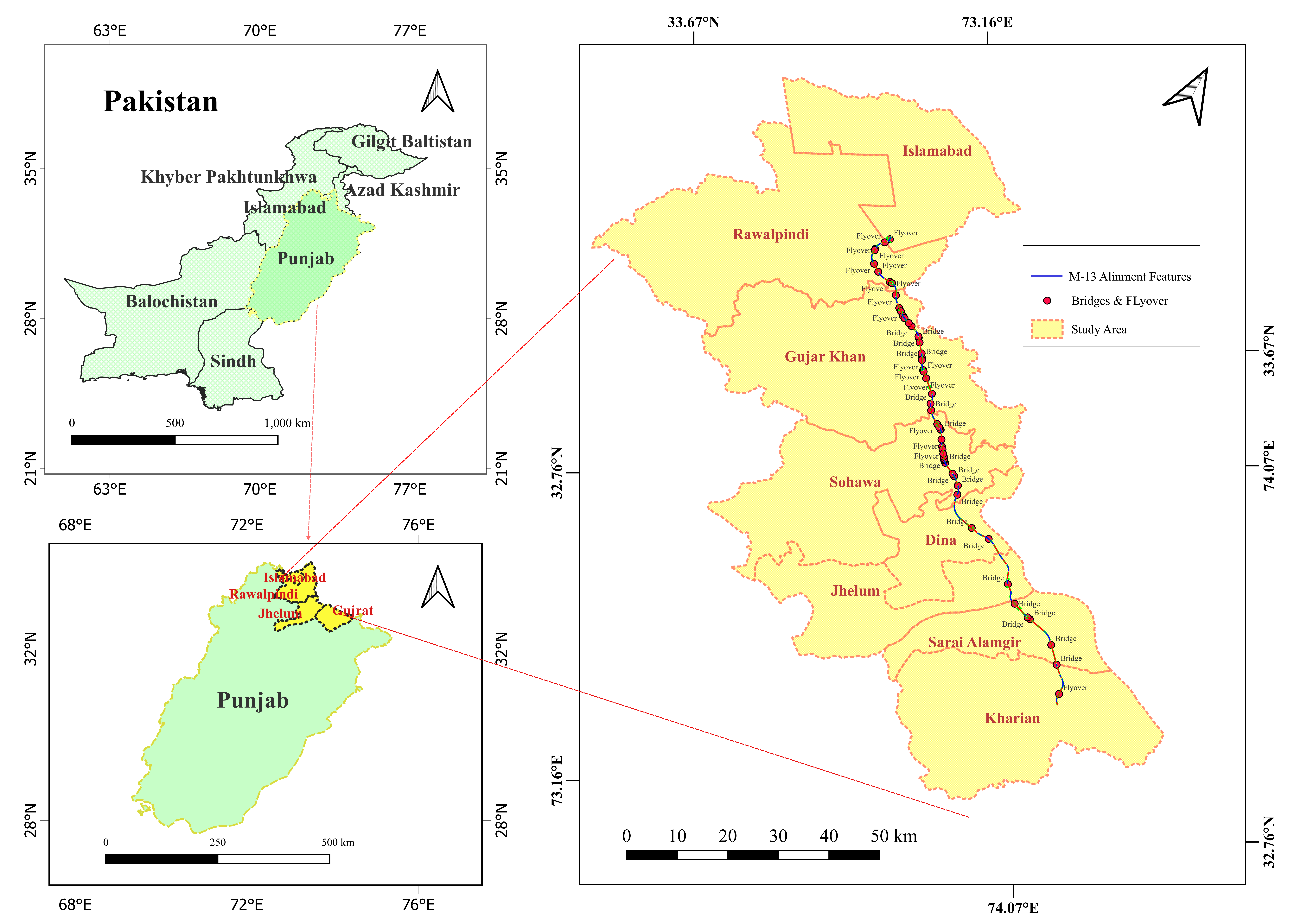

Supplement: Supplementary 1 — Figs. S1 to S6 Data S1 to S9 [file research.1175.f1.zip › Fig. S1.png]

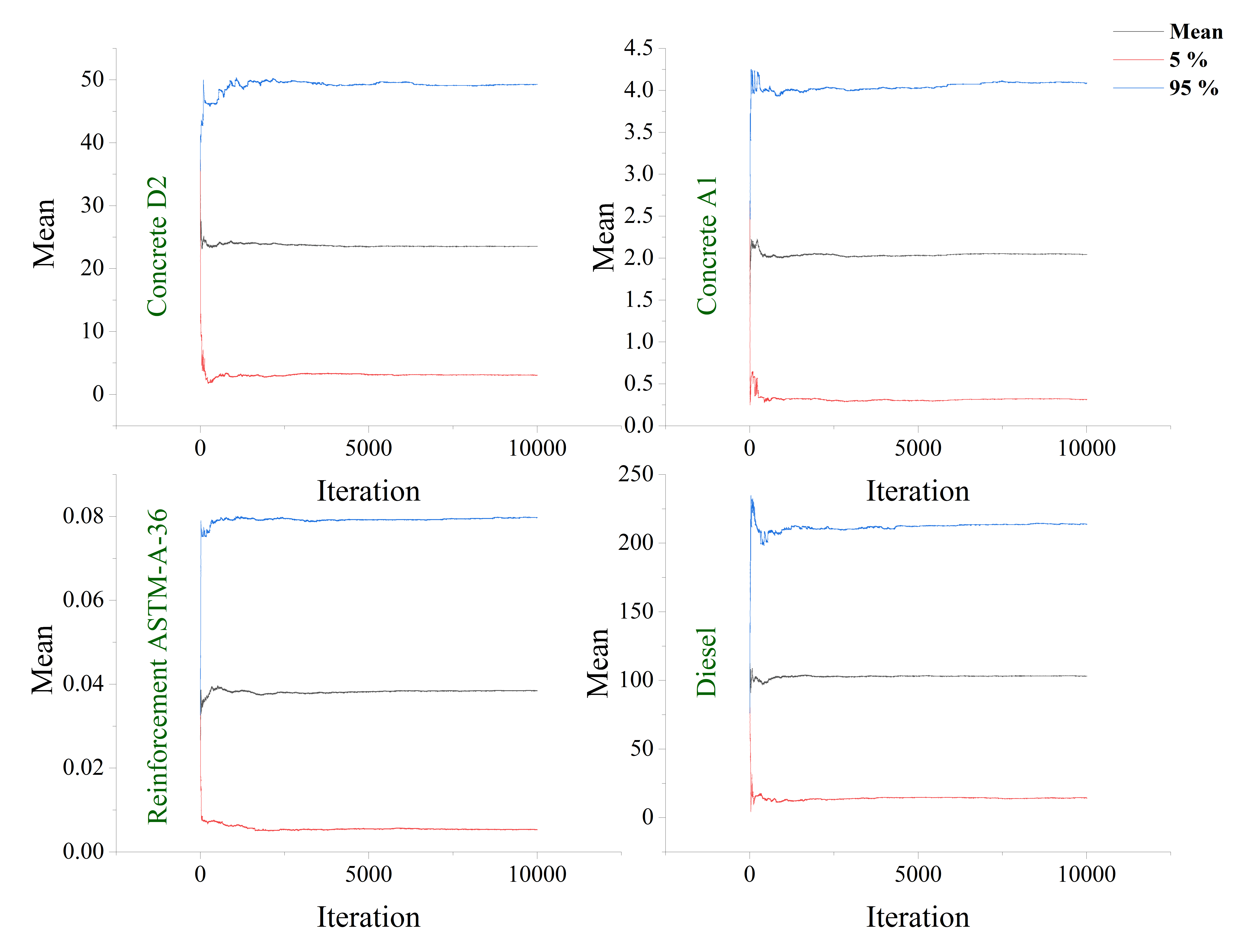

Supplement: Supplementary 1 — Figs. S1 to S6 Data S1 to S9 [file research.1175.f1.zip › Fig. S4.jpg]

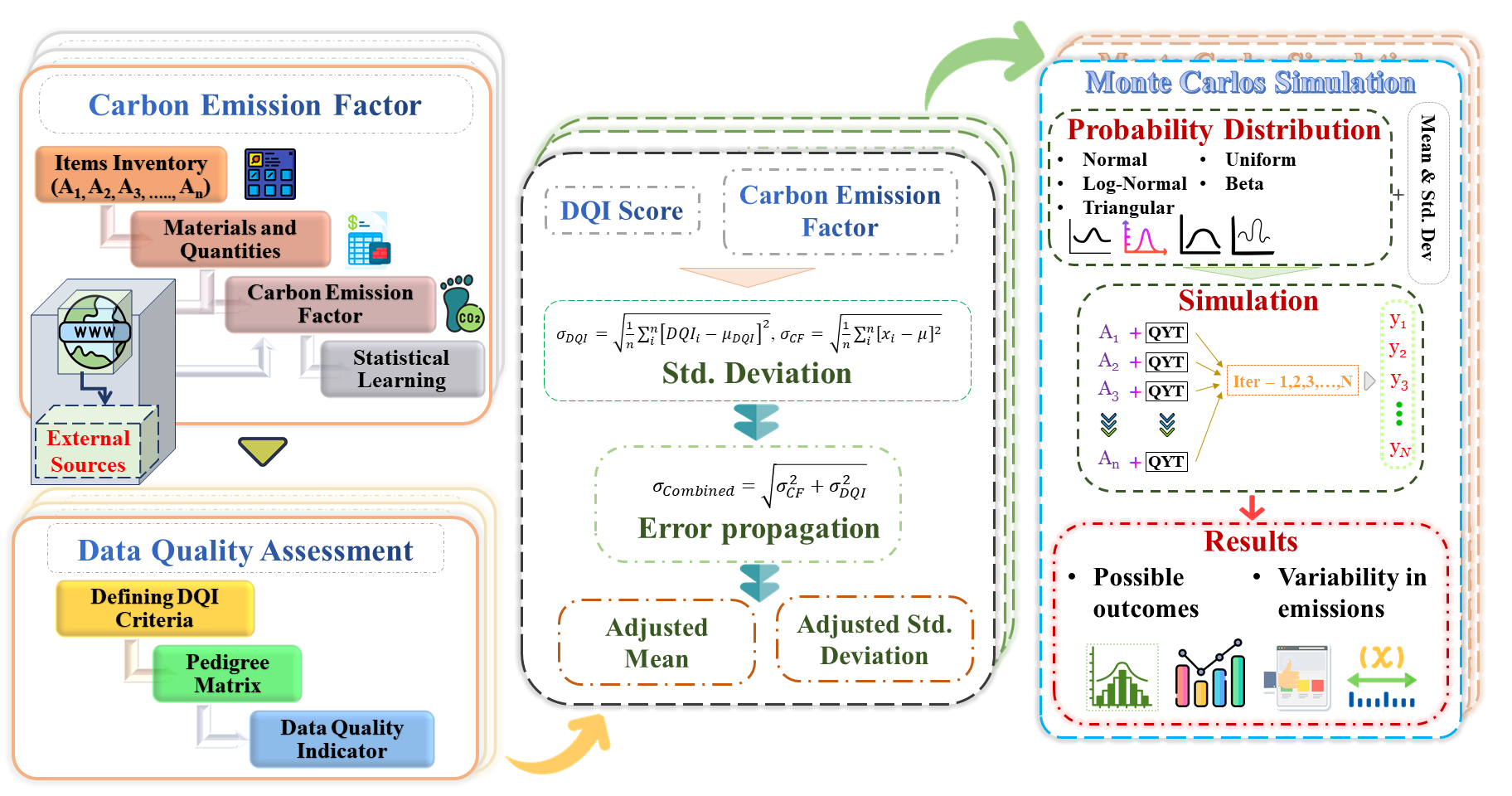

Supplement: Supplementary 1 — Figs. S1 to S6 Data S1 to S9 [file research.1175.f1.zip › Fig. S5.png]

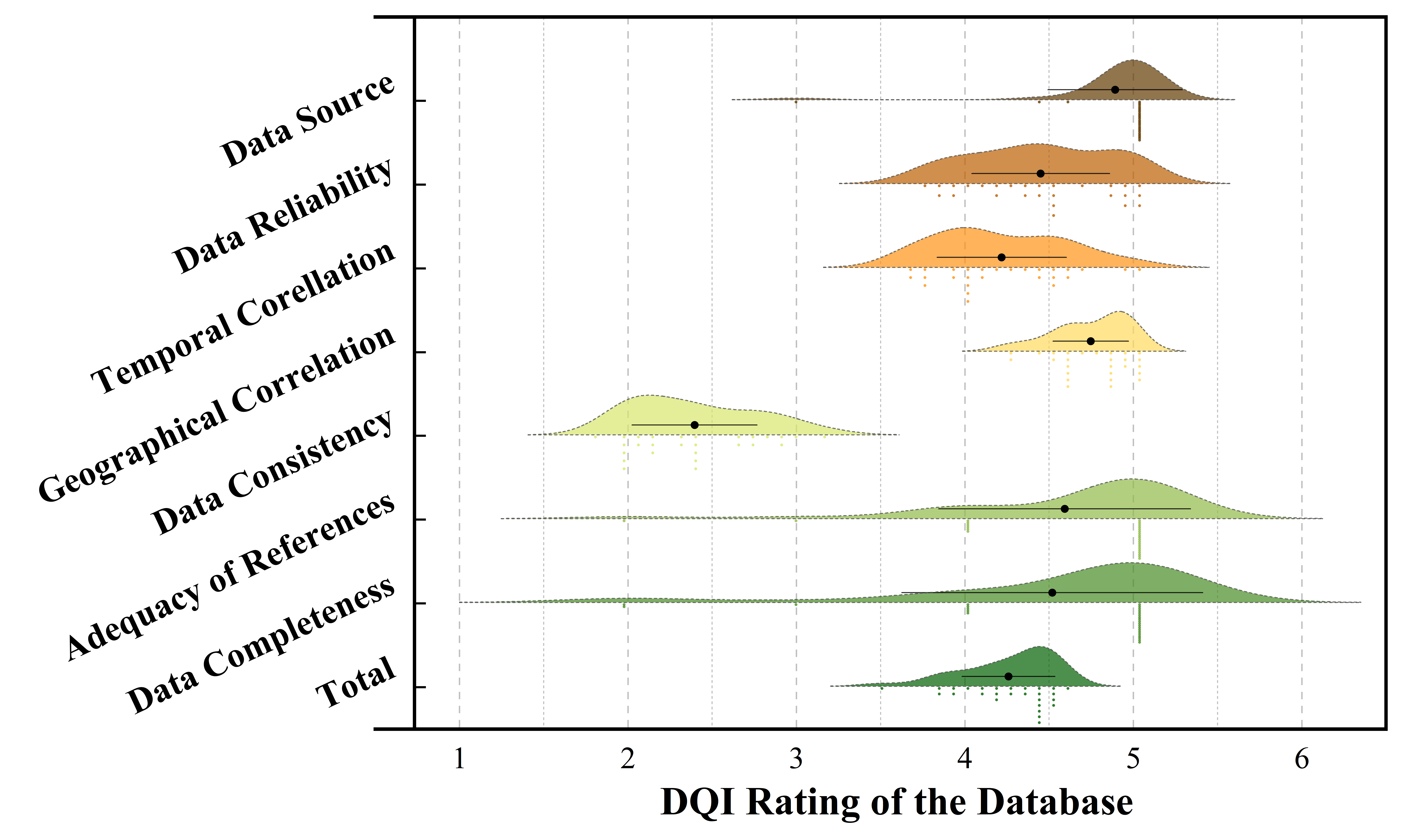

Supplement: Supplementary 1 — Figs. S1 to S6 Data S1 to S9 [file research.1175.f1.zip › Fig. S6.jpg]
